# Bioorthogonal catalytic nanozyme-mediated lysosomal membrane leakage for targeted drug delivery

**DOI:** 10.7150/thno.66325

**Published:** 2022-01-01

**Authors:** Zhiyuan Sun, Qiqi Liu, Xinyue Wang, Jin Wu, Xueyan Hu, Miaomiao Liu, Xiangyun Zhang, Yonghua Wei, Zhijun Liu, Hongjiang Liu, Rui Chen, Fei Wang, Adam C. Midgley, Aitao Li, Xiyun Yan, Yanming Wang, Jie Zhuang, Xinglu Huang

**Affiliations:** 1 College of Pharmacy, and State Key Laboratory of Medicinal Chemical Biology, Nankai University, Tianjin 300350, China.; 2 Key Laboratory of Bioactive Materials for the Ministry of Education, College of Life Sciences, Nankai University, Tianjin 300071, China.; 3 School of Medicine, Nankai University, Tianjin 300071, China.; 4 School of Materials Science and Engineering, Nankai University, Tianjin 300350, China.; 5 State Key Laboratory of Biocatalysis and Enzyme Engineering, Hubei Key Laboratory of Industrial Biotechnology, School of Life Sciences, Hubei University, Wuhan 430062, China.; 6 Joint Laboratory of Nanozymes, College of Life Sciences, Nankai University, Tianjin 300071, China.; 7 CAS Engineering Laboratory for Nanozymes, Institute of Biophysics, Chinese Academy of Sciences, Beijing 100101, China.

**Keywords:** Nanozyme, Lysosome, Bioorthogonal catalysis, Pro-drug, Targeted delivery, Tumor

## Abstract

**Rationale:** Employing *in situ* bioorthogonal catalysis within subcellular organelles, such as lysosomes, remains a challenge. Lysosomal membranes pose an intracellular barrier for drug sequestration, thereby greatly limiting drug accumulation and concentrations at intended targets. Here, we provide a proof-of-concept report of a nanozyme-based strategy that mediates *in situ* bioorthogonal uncaging reactions within lysosomes, followed by lysosomal escape and the release of uncaged drugs into the cytoplasm.

**Methods:** A model system composed of a protein-based nanozyme platform (based on the transition metals Co, Fe, Mn, Rh, Ir, Pt, Au, Ru and Pd) and caged compound fluorophores was designed to screen for nanozyme/protecting group pairings. The optimized nanozyme/protecting group pairing was then selected for utilization in the design of anti-cancer pro-drugs and drug delivery systems.

**Results:** Our screening system identified Pd nanozymes that mimic mutant P450_BM3_ activity and specifically cleave propargylic ether groups. We found that the intrinsic peroxidase-like activity of Pd nanozymes induced the production of free radicals under acid conditions, resulting in lysosomal membrane leakage of uncaged molecules into the cytoplasm. Using a multienzyme synergistic approach, our Pd nanozymes achieved *in situ* bioorthogonal catalysis and nanozyme-mediated lysosomal membrane leakage, which were successfully applied to the design of model pro-drugs for anti-cancer therapy. The extension of our nanozyme system to the construction of a liposome-based “all-in-one” delivery system offers promise for realizing efficacious *in vivo* tumor-targeted therapies.

**Conclusions:** This strategy shows a promising new direction by utilizing nanotechnology for drug development through in situ catalyzing bioorthogonal chemistry within specific subcellular organelles.

## Introduction

Lysosomes are membrane-bound subcellular organelles with an acidic interior, which contains at least 60 hydrolases for the degradation and recycling of essential nutrients, and maintainence of cell homeostasis. Growing evidence has implicated the roles of lysosomes in the onset of various pathologies, such as cancers 1, 2. Many cancer cells have been shown to have an increased number of lysosomes, which assist in the facilitation of cell proliferation and survival under microenvironmental stress 3. Additionally, lysosomes play important roles as mediators of drug resistance in cancers 4. Thus, lysosomes have been recently considered as novel targets for cancer treatment. For example, various pro-drugs have been successfully designed for anticancer therapy that respond to the lysosomal microenvironment, which has a low pH 5 and an abundance of hydrolases 6 and reactive oxygen species (ROS) 7. However, developing lysosome-targeting drugs remains in its infancy, for at least two crucial obstacles are evident. First, lysosomal drug sequestration - a common phenomenon of drug entrapment in lysosomes - markedly reduces drug concentrations in their intended targets (i.e. nucleus and cytoplasm) 8-10. Second, lysosome-targeting drugs exhibit a tendency to result in adverse side effects due to the incapabilities of current drug delivery strategies to distinguishing between lysosomes of healthy cells and those of tumor cells 2.

In recent years, bioorthogonal catalysis has emerged as a powerful tool for biomedical applications; arising from the ability to generate therapeutic agents *in situ*, which minimizes off-target effects 11-18. The implementation of this class of chemistry is mainly dependent on abiotic transitional metals 19-21, including Pd, Ir, Ru, Au and Cu. Two types of transition metal species, metal complexes and metal nanoparticles, have successfully been applied in cross-coupling and bioorthogonal unmasking reactions (BUR) for biomolecular labelling and pro-drug activation. The application of metal complexes has been greatly limited to bacterial and cellular studies due to their unpredictable cytotoxicity and uncontrollable stability. More importantly, it is particularly difficult to achieve metal complex-mediated BUR in subcellular organelles, such as lysosomes, primarily due to free diffusion of transition metals throughout the whole cell.

**Scheme 1:**
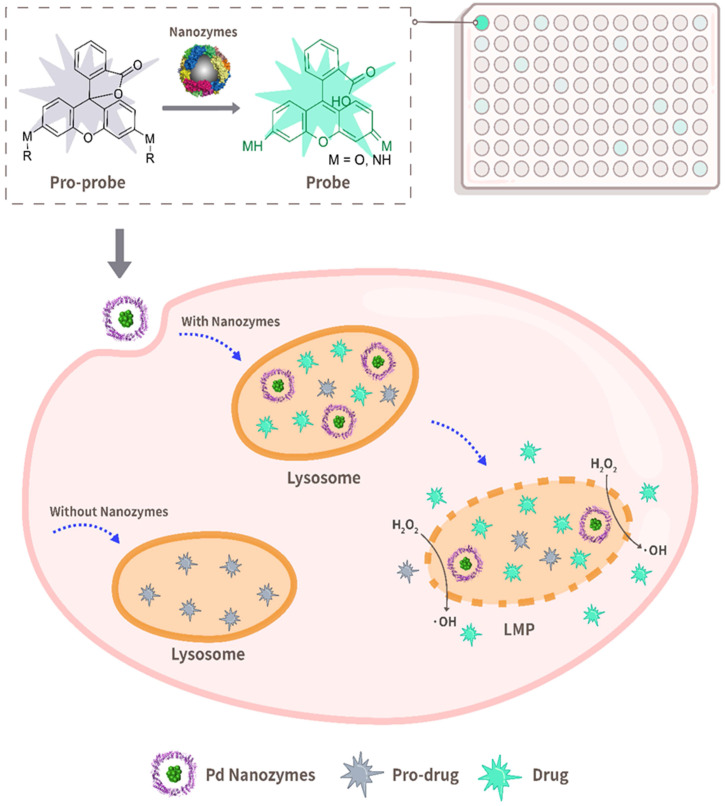
** Schematic illustration of bioorthogonal catalytic nanozyme-mediated lysosomal membrane leakage for targeted drug delivery.** A model system that was composed of a protein-based nanozyme platform (based on the transition metals Co, Fe, Mn, Rh, Ir, Pt, Au, Ru and Pd) and various caged compound fluorophores was successfully established for screening nanozyme/protecting group pairings. The obtained Pd nanozyme/ propargyl ethers pairing was further utilized for designing anti-cancer pro-drugs, capable of overcoming lysosomal drug sequestration and improving drug concentrations in their intended targets following pro-drug activation under Pd nanozyme catalysis.

In contrast to the free diffusion of metal complexes within cells, nano-sized particles are capable of being trapped within lysosome *via* passive and/or active endocytosis. Nanozymes are nanomaterials with enzyme-like characteristics. Nanozymes represent a new kind of artificial enzyme that exhibit the unique physicochemical properties of nanomaterials with enzyme-like catalytic activity, including but not limited to peroxidase (POD)-like activity 22-30. Importantly, most reported nanozymes contain transition metal elements. Thus, we hypothesized that nanozymes could regulate lysosomal BUR *in situ* for potential lysosome-targeting pro-drug development. However, it is extremely difficult to predict what kind of nanozyme is capable of catalyzing what kind of BUR. Herein, we established a model system that was composed of a protein-based nanozyme platform (based on the transition metals Co, Fe, Mn, Rh, Ir, Pt, Au, Ru and Pd) and caged compound fluorophores for screening nanozyme/protecting group pairings. To construct a nanozyme library, various transition metal nanoparticles were incorporated *in situ* into protein scaffolds. The caged compounds were tailored with model fluorophores (i.e. fluorescein or rhodamine) and various protecting groups. The system offers an ideal platform for paired screening of the corresponding cleaved bonds during nanozyme catalysis. The selected nanozyme/protecting group pairing provided a basis for the design of lysosome-relevant pro-drugs in anti-cancer therapy. Furthermore, the nanozymes entrapped within lysosomes facilitated the cleavage of the pro-drugs and induced lysosomal membrane leakage, which in turn promoted the diffusion of the cleaved drugs into the cytoplasm and their intended targets (**Scheme 1**).

## Materials and Methods

Additional materials and methods are provided in the Supplementary Materials, including the synthesis and characterization of compound F1-F9, compound 1ʼ-4ʼ, and pro- hydroxycamptothecin (HCPT).

### Synthesis of ferritin nanocages (FTn)-based nanozymes

Prior to preparation of nanozymes, FTn were purified according to our previously reported procedures 31, 32. The FTn-based nanozymes were prepared by *in situ* growth of metal nanoclusters into the FTn cavity. In a typical reaction, 2 mg FTn was added into 10 mL PBS buffer, and adjusted pH to 7.5-8.0. Then, 3.83 µmol K_2_PdCl_4_ was added into the solution and stirred at 60 °C for 1 h. The solution was purified using a PD-10 desalting column (GE Healthcare) to remove unincorporated metal ions. Sodium borohydride (NaBH_4_) was slowly added to the solution at ice bath and the resulting solution was further purified to obtain the Pd nanozymes using a PD-10 column. For the preparation of different sized Pd nanozymes, various amounts of K_2_PdCl_4_ (1.915-3.83 µmol) were added into 2 mg FTn solution. To synthesize different metal nanozymes (*i.e.* Pt-, Rh-, Au-, Ir- and Ru-nanozymes), the metal precursors such as K_2_PtCl_4_, RhCl_3_·3H_2_O, K[AuCl_4_]·2H_2_O, IrCl_3_ and RuCl_3_ were used to replace K_2_PdCl_4_ in a typical procedure.

MnO_2_ nanozyme was synthesized following protocols according to our previous study 33. Typically, 2 mg FTn was added into 10 mL degassed NaCl solution (100 mM) under N_2_ conditions. After adjustment pH to 8.5, 7.84 µmol MnCl_2_ was added into the solution kept at 60 °C by use of a water bath. The freshly prepared degassed H_2_O_2_ (H_2_O_2_: metal ion, 1:2) was added as an oxidant for the reaction. The constant pH was dynamically monitored using 50 mM NaOH. After finishing the reaction, sodium citrate (300 mM) was added to chelate free metal ions. The resulting solution was further purified to obtain the nanozyme using a PD-10 desalting column. Fe- and Co-nanozyme were synthesized by dissolving (NH_4_)_2_Fe(SO_4_)_2_·6H_2_O and CoCl_2_·6H_2_O into FTn solution reaction system following the same procedure.

FTn was characterized by transmission electron microscopy (TEM) (JEOL JEM-2800) following negative staining of the specimen with 1% uranyl acetate. The morphology of FTn nanozymes was characterized by TEM without negative staining. The protein concentration of FTn was determined with standard Bicinchoninic Acid (BCA) assay kit. The concentration of FTn-based nanozyme was determined by SDS-PAGE quantification of protein concentration using standard FTn samples. Pd nanozymes were characterized by energy dispersive spectroscopy (EDS, JEOL JEM-2800F) and X-ray photoelectron spectroscopy (XPS, Axis Ultra DLD). Pd elements involved in Pd nanozymes were quantified with inductively coupled plasma mass spectrometry (ICP-MS) (SpectroBlue).

### Purification of Mutant cytochrome P450 enzymes (P450_BM3_)

Mutant P450_BM3_ was purified following previously reported procedures 34. Briefly, the plasmid vector encoding mutant P450_BM3_ (mutations R47T/S72F/A82F/F87I/L437S) was transformed into competent *E. coli* BL21 (DE3). Isopropyl b-D-1-thiogalactopyranoside (IPTG) was added to a final concentration of 0.2 mM to induce the protein production. Cell lysate, prepared by sonication of the bacterial culture, was centrifuged for 30 min at 9,000 rpm at 4 °C to collect supernatant. Then the obtained brownish-red supernatant was filtered with a 0.45 μm filter. For further purification, the obtained lysate was loaded onto a nickel affinity column (GE Healthcare) and washed with 500 mM NaCl and 50 mM phosphate buffer (pH 8.0) containing different concentration of imidazole solution (10 - 250 mM). The protein was concentrated with Amicon Ultra centrifugal filters (cut off 50 kDa) for further experiments. The protein concentration of P450_BM3_ was determined with standard BCA assay kit.

### Screening of nanozyme/protecting group pairings

The screening of the nanozyme/protecting group pairings was performed in a 100 µL PB buffer by paired incubation of 150 nM various nanozymes with 20 µM F1-F9. After incubation for 45 min at 37°C, the fluorescence intensity (Ex = 450 nm, Em = 520 nm) was measured using a Molecular Devices Spectra Max M2 microplate reader (Multiskan FC, Thermo Fisher Scientific). Quantification analysis of fluorescence changes was determined by the increased fold of signal intensity. The fluorescence intensity of the compounds without nanozymes was normalized to 1.

For evaluation of F1-F9 activation by mutant P450_BM3_, 150 nM mutant P450_BM3_ (0.5 mM NADPH was added as co-substrate) and 20 µM F1-F9 were added into a 100 µL KPi buffer solution. The fluorescence intensity was determined following the above procedure.

### Evaluation of POD-like activity of Pd nanozymes

The POD-like activity of Pd nanozymes was investigated using tetramethylbenzidine (TMB) as the substrate in the presence of H_2_O_2_. Typically, the kinetic assay was performed in 0.1 M HAc-NaAc buffer solution by adding 150 nM Pd nanozymes, 0.44 M H_2_O_2_ and different concentration of TMB (0 - 4.16 mM). All reactions were recorded by measuring the absorbance of oxidized TMB at 650 nm, and the Michaelis-Menten constant was calculated according to the Michaelis-Menten saturation curve using GraphPad Prism v8.0.

To assess ·OH production of Pd nanozymes, hydroxyphenyl fluorescein (HPF) was used as an indicator. In a typical reaction, different concentration of Pd nanozymes (0 - 0.6 μM) were added into HAc-NaAc buffer (pH 5.0) containing 0.44 M H_2_O_2_ and 5 μM HPF. After incubation for 1 h, the fluorescence signal intensity was recorded with a microplate reader at 515 nm.

### Fluorescent labeling of FTn

For fluorescent labeling, Cy5.5-NHS ester or Cy5-NHS ester was reacted with FTn (molar ratio, 50:1) at room temperature for 4 h in PBS solution (pH 8.0). The resulting mixture was purified with a PD-10 desalting column (GE Healthcare). The number of Cy5/Cy5.5 per FTn was determined by measuring the concentration of FTn and Cy5/Cy5.5, respectively. The Cy5.5/Cy5 concentration was determined using the absorbance value (Cy5 at 640 nm, Cy5.5 at 680 nm) and the established standard curve of Cy5.5/Cy5 sample.

### High performance liquid chromatography (HPLC) analysis

The reactions were performed by dissolving 1 µmol compound 1'-4' in 1 mL ultra-pure water in the presence or absence of 32 nmol Pd nanozymes and incubation at 37 ^o^C for 12 h. The conversion rate of compounds 1'-4' was investigated by HPLC. Absorbance at 280 nm was monitored for compounds 1' and 2'. For compounds 3' and 4', absorbance at 330 nm was observed. HPLC conditions for analysis of reaction products in methanol: 0.1% trifluoroacetic acid (TFA)/H_2_O (70-30 at 0-1 min; 50-50 at 1-11 min; 40-60 at 11-16 min; 30-70 at 16-21 min, and 70-30 at 21-30 min), with a flow rate of 0.8 mL/min.

### Preparation of compounds or nanozymes loaded Liposome

To obtain F1 or pHCPT loaded liposomes, the mixture of 1,2-dioleoyl-sn-glycero-3-phosphocholine (DOPC), cholesterol, 1,2-distearoyl-sn-glycero-3-phosphoethanolamine-N-[methoxy(polyethylene glycol)-2000] (Ammonium Salt) (DSPE-PEG_2k_) or DSPE-PEG_2k_-RGD (RGD: DSPE-PEG_2k_ at a 10:1 molar ratio), and F1 or Pro-HCPT molecules at a molar ratio of 9:4.5:0.6:1 were dissolved in chloroform and then dried under a rotary evaporator. Afterward, the dried lipid film was hydrated with PBS, followed by extrusion through 400 nm, 200 nm and 100 nm polycarbonate filters a total of 20 times before further use.

For loading Pd nanozymes into Lipo-F1 or Lipo-pHCPT, the obtained lipid film of Lipo-F1 or Lipo-pHCPT was fully hydrated with 1 mL solution at 60 °C for 0.5 h containing Pd nanozymes at a concentration of 2 mg/mL. Then the mixture was subjected to five freeze-thaw cycles (5 min in liquid nitrogen and 5 min at 60 °C) to make sure Pd nanozymes loaded into Lipo-F1 or Lipo-pHCPT. After removing the free Pd nanozymes and free F1/pro-HCPT by gradient centrifugation, the obtained pellet was resuspended with PBS. The resulting solution was sequentially extruded through 400 nm, 200 nm and 100 nm polycarbonate filters before further use.

### Stability of Lipo-Pd-pHCPT

The stability of Lipo-Pd-pHCPT at different pH was investigated by measuring fluorescent emission spectrum of pHCPT. Briefly, Lipo-Pd-pHCPT (pHCPT equivalent, 300 µM) was incubated with different pH buffers (i.e. pH 7.4 and 5.0) at 37 ^o^C. After 24 h incubation, the fluorescent emission were measured (Ex = 360 nm) using a Molecular Devices Spectra Max M2 microplate reader. The changes of fluorescent emission peak determined the activation of pHCPT.

### Cell culture

Human breast cancer cells (MDA-MB-231), human brain glioblastoma cells (U87), rat cardiac myoblasts (H9C2) and mouse embryonic fibroblasts (NIH 3T3) were cultured in high glucose DMEM (HyClone) supplemented with 10% fetal bovine serum and 1% Penicillin-Streptomycin. All cells were incubated under normal conditions in a humidified incubator (37 ^o^C, 5% CO_2_).

### Lysosomal membrane permeability (LMP) assay

LMP assay was assessed by acridine orange (AO) staining. The AO produces red fluorescence (Ex/Em 488/640 nm) when accumulated within acidic lysosomes, and distributes throughout the cytoplasm as green fluorescence (Ex/Em 488/525 nm) in response to LMP. After 320 nM Pd nanozymes treatment, the MDA-MB-231 cells were stained with AO (38 nM) at 37 ^o^C for 20 min. For ascorbic acid (AA) inhibitor assay, 0.4 mM AA and 320 nM Pd nanozymes were simultaneously added to MDA-MB-231 cells. Then the cells were imaged using a Zeiss confocal laser scanning microscope with Ex/Em 488/525 nm and Ex/Em 488/640 nm wavelengths. The intracellular LMP was determined by quantification analysis of the ratio of red fluorescence in lysosomes and green red fluorescence in cytoplasm and nucleus.

### F1 activation by Pd nanozymes in cells

To evaluate F1 activation *in vitro*, MDA-MB-231 cells were seeded into glass-bottom dishes (1 × 10^5^ cells per well) and cultured for 24 h, respectively. The cells were incubated with or without Pd nanozymes for 4 h, and then incubated with F1 (20 µM) or Lipo-F1 (F1 equivalent, 20 µM) for another 4 h, followed by staining with LysoTracker Red (100 nM, Beyotime) at 37 ^o^C for 30 min and Hoest3342 (Beyotime) at 37 °C for 10-20 min. For ketoconazole (KET) inhibitor assay, 30 µM KET and 20 µM F1 were added following MDA-MB-231 cells treatment with Pd nanozymes. The activation of F1 in cells was observed by using Zeiss confocal microscopy with Ex/Em 488/520 nm for F1, Ex/Em 577/590 nm for LysoTracker Red and Ex/Em 633/670 nm for Cy5-labeled Pd nanozymes, respectively. The signal intensity of the particles in cells was quantified by Image J software.

### Cytotoxicity of pro-HCPT *in vitro*

Cytotoxicity was measured using standard methyl thiazolyl tetrazolium (MTT) assay. Briefly, the cells were seeded into 96-well plates (4 × 10^3^ cells per well). After 24 h, pro-HCPT (0-10 μM) were added into the cells with or without Pd nanozymes (160 nM). After 48 h incubation, cytotoxicity was assessed with a standard MTT method. The cell relative activities were calculated as OD_570_ mean value in experimental groups/OD_570_ mean value in control group × 100%.

### *In vivo* and *ex vivo* tumor imaging

For tracking distribution of liposome *in vivo*, mice bearing MDA-MB-231 tumors were administered with FTn-Cy5.5 loaded Liposome-based formulations *via* tail vein injection (FTn equivalent of 50 mg/kg). At the indicated time points, the fluorescence imaging was acquired using a Xenogen IVIS Spectrum imaging system (IVIS Lumina II Xenogen, Caliper Life Sciences). The optimal imaging parameters of the particles were determined based on the specific signal intensity in mouse body obtained from the imaging system. For analyzing the biodistribution, the mice were sacrificed and major organs including heart, liver, spleen, lung, kidney, muscle, and tumor were collected for imaging. The accumulation and distribution of the particles were semi-quantified by using the Living Image 2.50 software. For the distribution of the particles in tumor tissues, the Cy5-labeled particles were administrated via tail vein into the mice bearing MDA-MB-231 tumors. The tumors were harvested and embedded in optimal cutting temperature (OCT) compound. The tumor tissues were then sectioned at a thickness of 10 μm and a series of sections were immunostained with anti-CD31-PE antibody (BioLegend). After staining with DAPI, the slices were imaged using Zeiss confocal microscopy with Ex/Em 405/461 nm for DAPI, Ex/Em 565/578 nm for CD31 and Ex/Em 633/670 nm for Cy5-labeled liposomes, respectively.

### Animals and tumor model

All animal studies were approved by the Animal Ethics Committee of Nankai University and followed the Tianjin Committee of Use and Care of Laboratory Animals guidelines. Female BALB/c-Nude mice (4-5 weeks old) were purchased from SPF Biotechnology (Beijing, China). In this experiment, the mice bearing MDA-MB-231 subcutaneous tumors were randomly divided into four groups: PBS, Pd nanozymes (8 mg/kg), Lipo-pHCPT (5 mg/kg) and Lipo-Pd-pHCPT (5 mg/kg pro-HCPT+8 mg/kg Pd nanozymes). When tumor volumes reached ~100 mm^3^, the mice were systemically administrated with nanoparticles. The tumor size and mice weight were measured every other day. Tumor size was calculated using the following equation: tumor size = (longest tumor diameter) × (smallest tumor diameter)^2^/2. For the Kaplan-Meier survival curve, mice were monitored until death or considered expired when the tumor volume reached 1500 mm^3^.

### Hematoxylin and eosin (H&E) staining

For H&E staining analysis, 14 days post-treatment, H&E staining of the major organs (heart, kidney, liver, spleen and lung) from different groups were performed following standard procedures. Briefly, the tissues were fixed in 4% paraformaldehyde in PBS overnight. After gradual dehydration, tissues were embedded into paraffin. The paraffin-embedded tissues were sectioned into slices with 5 μm thickness. The slices were then stained with H&E for histopathological examination.

### Statistical analysis

Two-tailed, unpaired Student's t-tests were used to compare statistical significance between two groups of independent data. If multiple comparisons were involved, one-way or two-way analysis of variance (ANOVA) followed by post-test Tukey analysis were used to determine statistical significance. A p-value < 0.05 was considered statistically significant. Data is presented as the mean ± standard deviation (SD).

## Results and Discussion

### A screening system based on nanozymes and compound fluorophores

**Figure 1:**
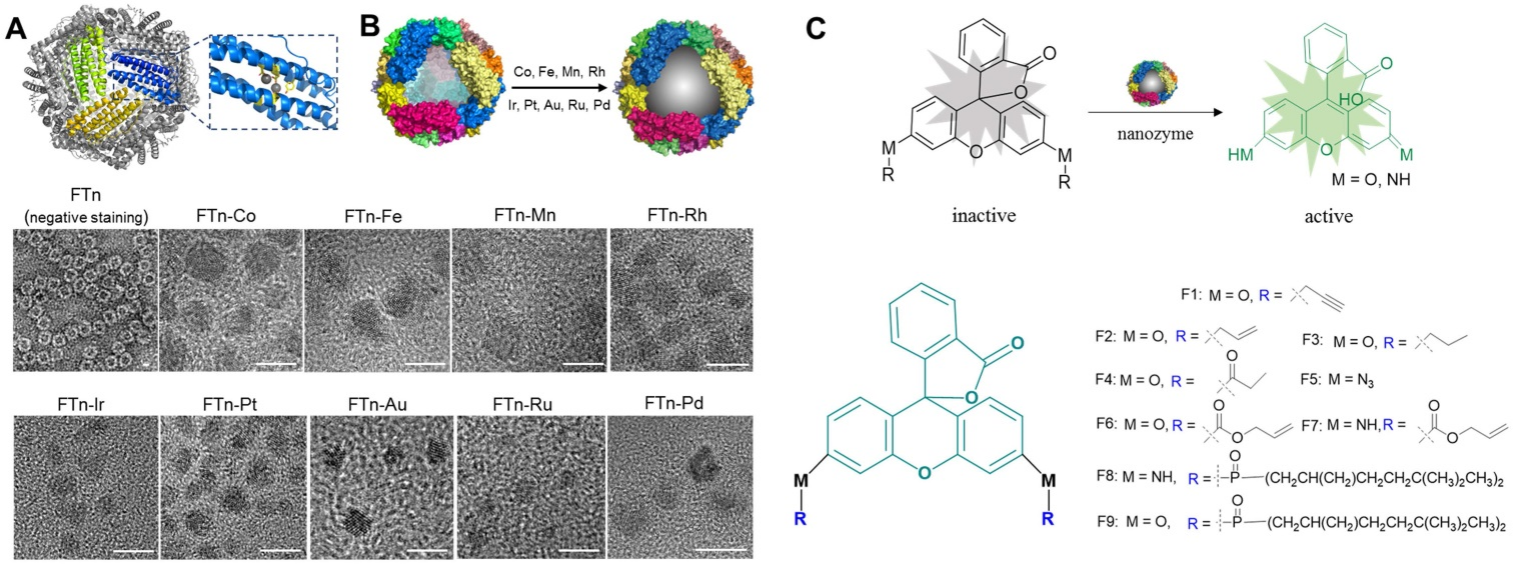
** The model screening system composed of FTn-based nanozymes and caged fluorophore compounds.** (A) Protein structure of FTn (PDB ID 3AJO). The colored domains indicate FTn subunits containing metal affinity region. The dark spheres represent the metal binding sites of FTn. (B) Schematic illustration (top) and TEM images (bottom) of various metal-based nanozymes. Top, a nanozyme library was established by *in situ* growth of various metal-based particles into FTn inner cavity. Bottom, TEM images of FTn and FTn-based nanozymes. For FTn imaging, the samples were firstly performed by negative staining of the specimen with 1% uranyl acetate. Scale bar = 5 nm. (C) Various fluorophore-based compounds by caging with different protecting groups. Top, schematic illustration of the compounds transtion from fluorescence “OFF” to fluorescence “ON” under nanozyme catalysis. Bottom, different protecting groups used to cage fluorophore and prevent fluorescence emission.

Our previous studies demonstrated that human FTn, assembled from 24 ferritin heavy chain subunits, were ideal artificial enzyme-mimicking carriers according to the intrinsic properties of the FTn protein scaffold 32, 35, which include channels for hydrophilic metal ion entry at the 3-fold symmetry axes and ferroxidase sites (Glu27, His65, Tyn 34, Glu 61, Glu107, Glu62, Gln141) for active metal ion binding (**Figure 1A**). We also showed that FTn protein scaffolds provide a location for *in situ* Mn^2+^ nucleation into MnO_2_ nanoparticles (i.e. Mn nanozymes), which possessed superoxide dismutase- and catalase-like activity 33. According to these studies, we herein developed a nanozyme-based platform by systematically employing *in situ* incorporation various transition metals into the FTn cavity, including Co, Fe, Mn, Rh, Ir, Pt, Au, Ru and Pd (**Figure 1B,** top). In other words, Co^2+^, Fe^2+^ and Mn^2+^ were oxidized to nucleate into metal oxides, whereas Rh^3+^, Ir^3+^, Au^3+^, Ru^3+^, Pt^2+^ and Pd^2+^ were reduced to form metal clusters. Protein negative staining and TEM showed the hollow structure of FTn nanoshells with 8 nm diameters (**Figure 1B,** bottom), while the *in situ* incorporation of metal nanoparticles into FTn nanoshells was directly observable by TEM. The mean diameters of the different metal oxides and metal clusters were measured to be approximately ~5 nm and ~2 nm, respectively.

**Figure 2:**
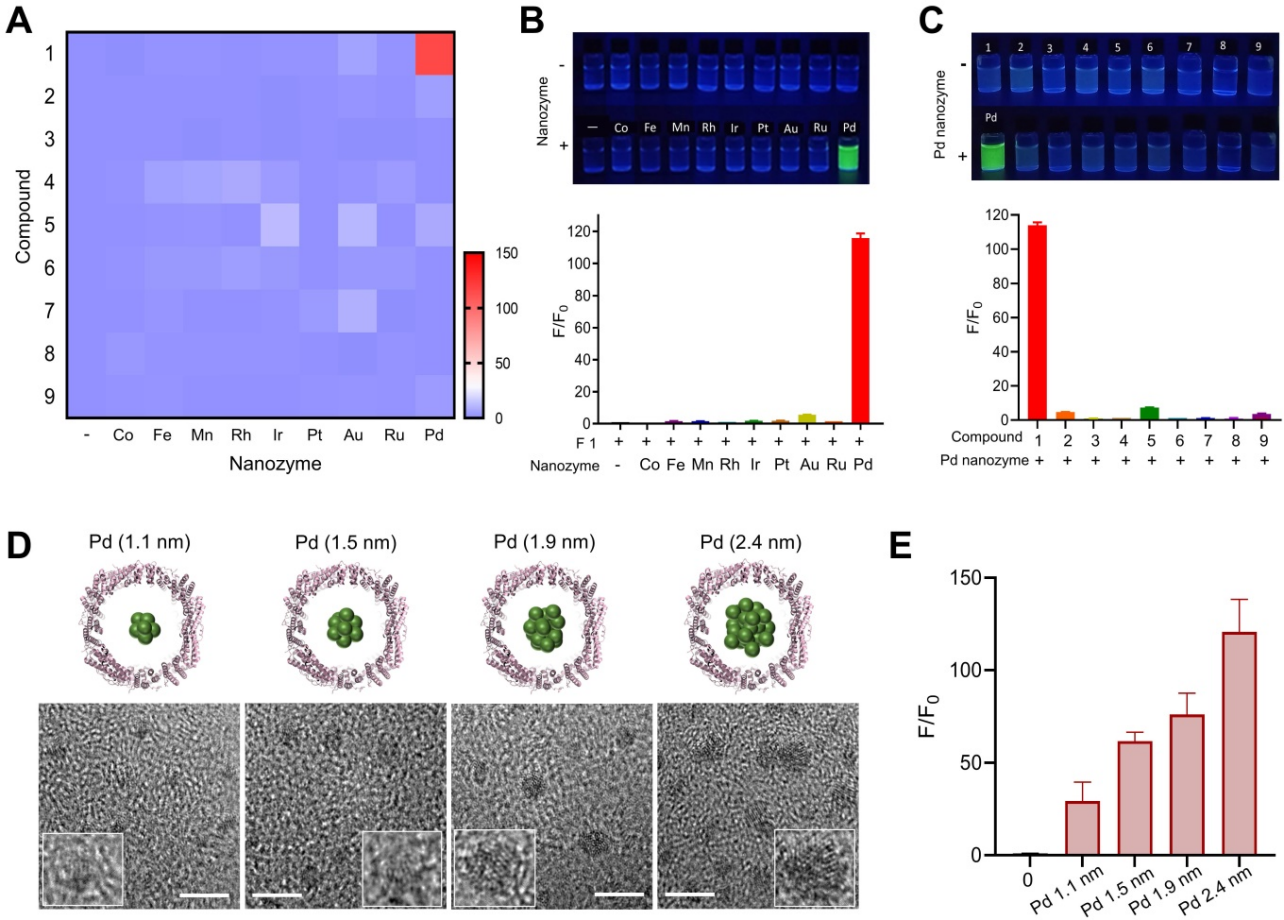
** Screening of nanozyme/protecting group pair.** (A) Heatmap of nanozyme activity towards different caged compounds in the established screening system. The fluorescence intensity of the compounds without nanozymes was normalized to 1. Scale bar represents the increased fold of fluorescence intensity. (B) Fluorescence changes of F1 in the presence of fixed concentration of various nanozymes and (C) Fluorescence changes of various compounds in the presence of Pd nanozymes under the optimal conditions. The nanozyme concentration was determined by measuring FTn protein concentration. Top, the fluorescence imaging of the compound solution incubated with or without different nanozymes under UV excitation. Bottom, quantification analysis of fluorescence changes of the compounds by the catalysis of various nanozymes. The fluorescence intensity of the compounds without nanozymes was normalized to 1. (D) TEM images of different sized Pd nanozymes integration into FTn inner cavity. Scale bar = 5 nm. (E) The effect of different sized nanozymes on F1 activation. The fluorescence intensity of F1 without nanozymes was normalized to 1.

Inspired by directed evolution of enzymes, we considered artificial nanozymes for the expansion of BUR by catalyzing the cleavage of different protecting groups. As model reactions, we designed various compound fluorophores 1-9 (F1-F9) by caging fluorophores with different protecting groups (**Figure 1C**). The detailed information on the synthesis and characterization of F1-F9 were provided in **Scheme S1, Figure S1 and S2**. Caged fluorophores were nonfluorescent but recovered their fluorescence upon uncaging. Using this system, the cleavage reaction could be detected in a straightforward fashion through fluorescence measurements. As such, we next sought to screen the nanozyme/protecting group pairings by paired incubation of the nanozymes with the compound fluorophores in a 96-well microplate. As shown in **Figure 2A**, heatmap data demonstrated the fluorescent signal changes of various compound fluorophores after nanozyme treatments at 37℃ for 45 min, compared to initial signal intensity. Interestingly, we found that only Pd nanozymes induced substantial increases of compound F1 fluorescence signal under this condition. The signal induction of other pairings were negligible, revealing that propargylic ether is specifically cleaved by Pd nanozymes in this screening system. To further confirm the specificity of Pd nanozymes/propargylic ether, the reactions were performed under optimal conditions using equal concentrations of F1 catalyzed with different nanozymes (**Figure 2B**), or defined concentrations of Pd nanozymes with different caged compounds (**Figure 2C**). In both cases, Pd nanozymes showed the highest activity for the cleavage of the propargylic ether bond of F1, which yielded a nearly 120-fold induction of fluorescence response. In view of specific cleavage of the propargylic ether bond by Pd nanozymes, we further characterized Pd nanozymes by EDS and XPS. The distribution of Pd element analyzed by EDS elemental mapping (**Figure S3A**) revealed a homogeneous dispersion of Pd element. XPS of the Pd nanozymes confirmed the chemical state of Pd by two characteristic peaks, displayed at 340.7 and 335.4 eV corresponding to the Pd (0) 2p3/2 and Pd (0) 2p5/2 spin-orbit peaks of Pd nanozymes, respectively (**Figure S3B**). The mechanism of Pd nanozyme cleavage of propargylic ether bonds was previosuly explained 34. Briefly, Pd element is present in its low-spin form in the Pd nanozymes. Molecular oxygen binds to the palladium and undergoes reductive scission, forming intermediate transition state with its oxidized Pd (II) and liberating the second oxygen atom as a water molecule. The hydrogen atom transferred from the substrate yields another intermediate transition state, which then transfers its hydroxyl group to the substrate radical. Then, the oxidized substrate dissociates and is replaced by water, returning the enzyme to its low-spin form Pd (0).

We next explored the effect of the sizes of Pd nanozymes on the cleavage of the propargylic ether bond of F1. Different sized nanozymes were prepared by adjusting the molar ratio of metal ions and FTn, and TEM images verified that various sized Pd nanoclusters were successfully obtained, with mean diameters of 1.1 nm, 1.5 nm, 1.9 nm and 2.4 nm, respectively (**Figure 2D**). After incubation of different sized Pd nanozymes of equivalent protein molar concentration with caged F1 compound, the fluorescence intensity of F1 increased to approximately 29-, 62-, 76-, 121-fold for nanozymes of 1.1 nm, 1.5 nm, 1.9 nm and 2.4 nm compared that of without Pd nanozymes, respectively (**Figure 2E**). These results implied that the catalytic capacity of Pd nanozymes was related to interfacial catalysis at the particle surface.

### Enzyme-mimicking activities

**Figure 3:**
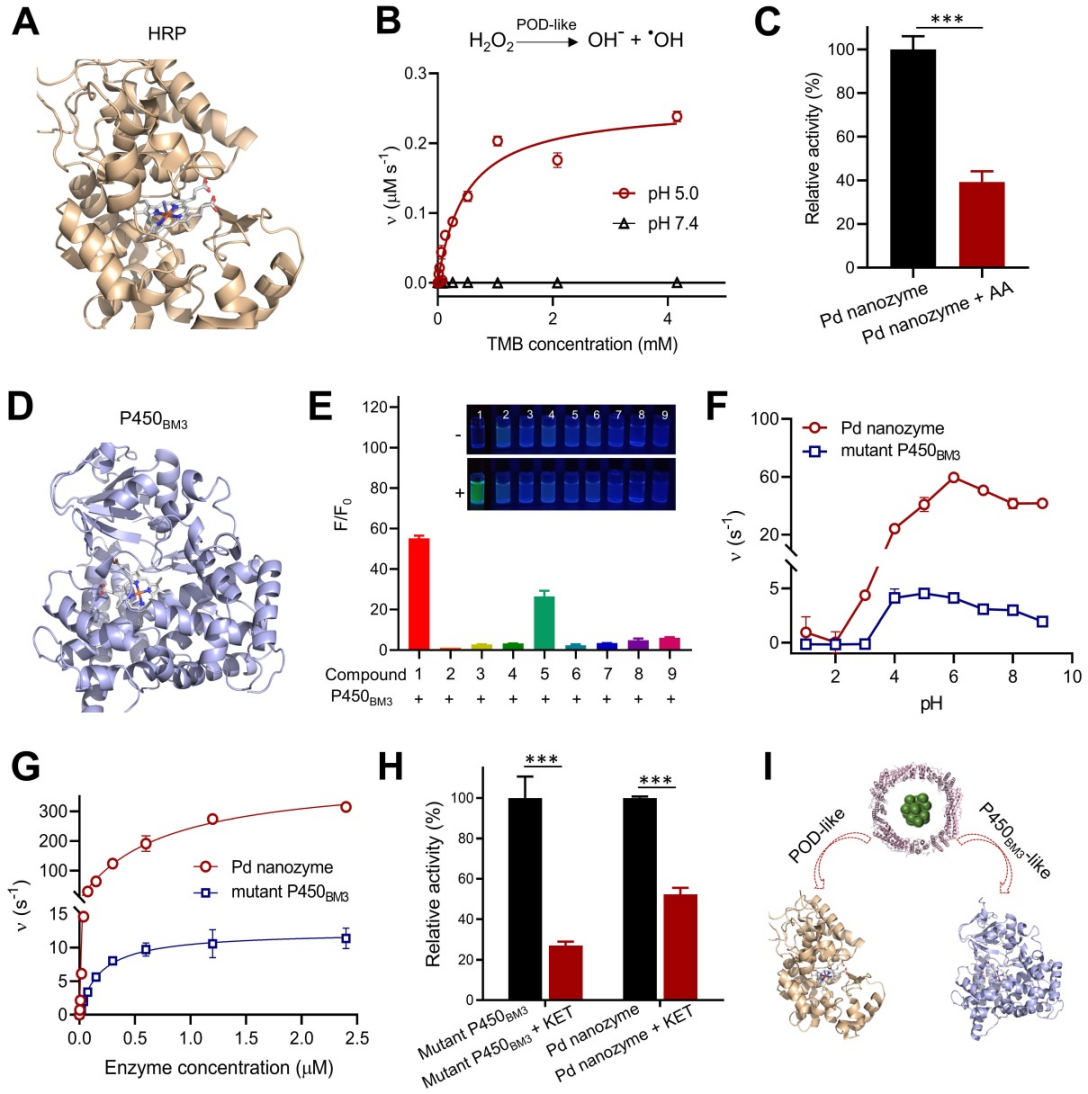
** Pd nanozymes possess POD-like and mutant P450_BM3_-like activities.** (A) Protein structure of HRP (PDB ID: 1W4Y). Blue represents the iron-containing catalytic active center of HRP. (B) POD-like activity of Pd nanozymes at pH 5.0 and pH 7.4. (C) Inhibition effect of AA inhibitors on POD-like activity of Pd nanozymes. *** *P* < 0.001. (D) Structure of P450_BM3_ (PDB ID: 4ZFA). P450_BM3_ variants indicate mutations R47T/S72F/A82F/F87I/L437S. Blue represents heme-iron active center. (E) Enzymatic activity of mutant P450_BM3_ towards caged compounds at fixed enzyme concentrations. (F) Enzymatic activity comparison of Pd nanozymes and mutant P450_BM3_ towards F1 at different pH. The observed fluorescence response for F1 was performed by the same molar concentration of Pd nanozymes and mutant P450_BM3_. The Pd nanozyme concentration was determined by measuring FTn protein concentration. (G) Enzymatic activity dynamics of Pd nanozymes and mutant P450_BM3_ towards F1 at different enzymatic concentration. (H) The competitive inhibition effect of KET on Pd nanozymes and mutant P450_BM3_. (I) Shematic illustration of Pd nanozymes with intrinsic POD-like and mutant P450_BM3_-like activities.

It is critical important to understand that nanozyme mimics what kind of native enzyme, facilitating to determine their inhibitors and further replace native enzyme for bioapplications. Previous reports demonstrated that almost all nanozymes possess POD-like activity under acidic conditions 36. We thus tested whether Pd nanozymes possessed horseradish peroxidase (HRP)-like activity by catalyzing the corresponding substrates (**Figure 3A**). As shown in** Figure 3B**, Pd nanozymes exhibited a high POD-like activity at pH 5.0 according to the analysis of typical Michaelis-Menten plot; in contrast, the POD-like activity of Pd nanozymes was negligible at neutral pH (pH 7.4). AA has been identified as an inhibitor of native POD through the antioxidant effect of AA, which efficiently suppresses the conversion of substrate H_2_O_2_ to hydroxyl-radicals 37. We also confirmed that POD-like activity of Pd nanozymes was substantially inhibited by AA under the same conditions (*P* < 0.001) (**Figure 3C** and **Figure S4A**).

In addition to POD-like activity, we sought to explore whether Pd nanozymes also possessed other enzyme-mimicking activities. As demonstrated in Figure 2, Pd nanozymes specifically cleaved the propargylic ether bond, and previous work demonstrated that the derivative of P450_BM3_ obtained by directed evolution technology efficiently induced the cleavage of propargylic ether 34 (**Figure 3D**). Given their catalytic cleavage of the same chemical bond (i.e. substrate), we thus sought to compare the properties of Pd nanozymes and mutant P450_BM3_. We first studied the specificity of the mutant P450_BM3_ for propargylic ether bond by determining the activation of the caged fluorophores, F1-F9. Consistent with the Pd nanozymes, the mutant P450_BM3_ also efficiently activated F1 (**Figure 3E**). At the identical protein molar concentration (Pd nanozymes concentration was determined with FTn protein concentration), we next compared the activity of Pd nanozymes and mutant P450_BM3_ by catalyzing F1 activation under various pH. We found the optimal pH for the Pd nanozymes was pH 5-9 (**Figure 3F**), whereas that of the mutant P450_BM3_ was pH 4-7. The Pd nanozymes maintained optimal activity over a wide range of temperatures, from 55 ^o^C to 65 ^o^C (**Figure S5**), and in accordance with the temperatures tolerance of FTn protein. In contrast, the mutant P450_BM3_ showed hightened activity only at physiological temperature (37 ^o^C). We further studied the enzymetic dynamics by using the typical Michaelis-Menten kinetic model (**Figure 3G**). The affinity of Pd nanozymes for F1 was comparable with mutant P450_BM3_ (**Table S1**), as evidenced by the *K*_m_ analysis. However, the catalytic efficiency of the Pd nanozymes was approximately 40-fold higher than mutant P450_BM3_, as evidenced by the values of *k*_cat_/*K*_m_. Quantification analysis using inductively coupled plasma mass spectrometry (ICP-MS) revealed approximately 120 Pd elements incorporated into each FTn protein, whereas each mutant P450_BM3_ includes only one heme-containing catalytic center 38. Thus, a possible explanation could be that the nanocluster structures of Pd nanozymes possess abundant catalytic active sites for interfacial catalysis at the particle surface, which resulted in greater catalytic activity than the haem-containing center of mutant P450_BM3_. Next, we studied enzyme activity in the presence of ketoconazole (KET), a well-known competitive inhibitor for P450_BM3_ that prevents pro-fluorescent F1 binding to the active site of Pd nanozymes. After addition of KET, the enzymatic activity of both mutant P450_BM3_ and Pd nanozymes were significantly inhibited at the identical conditions (**Figure 3H** and **Figure S4B**). Together, Pd nanozymes not only possessed POD-like activity but also exhibited mutant P450_BM3_-like activity (**Figure 3I**), and their corresponding inhibitors of enzymatic activities were also identified for further studies.

### Pd nanozyme-mediated lysosomal membrane leakage

**Figure 4:**
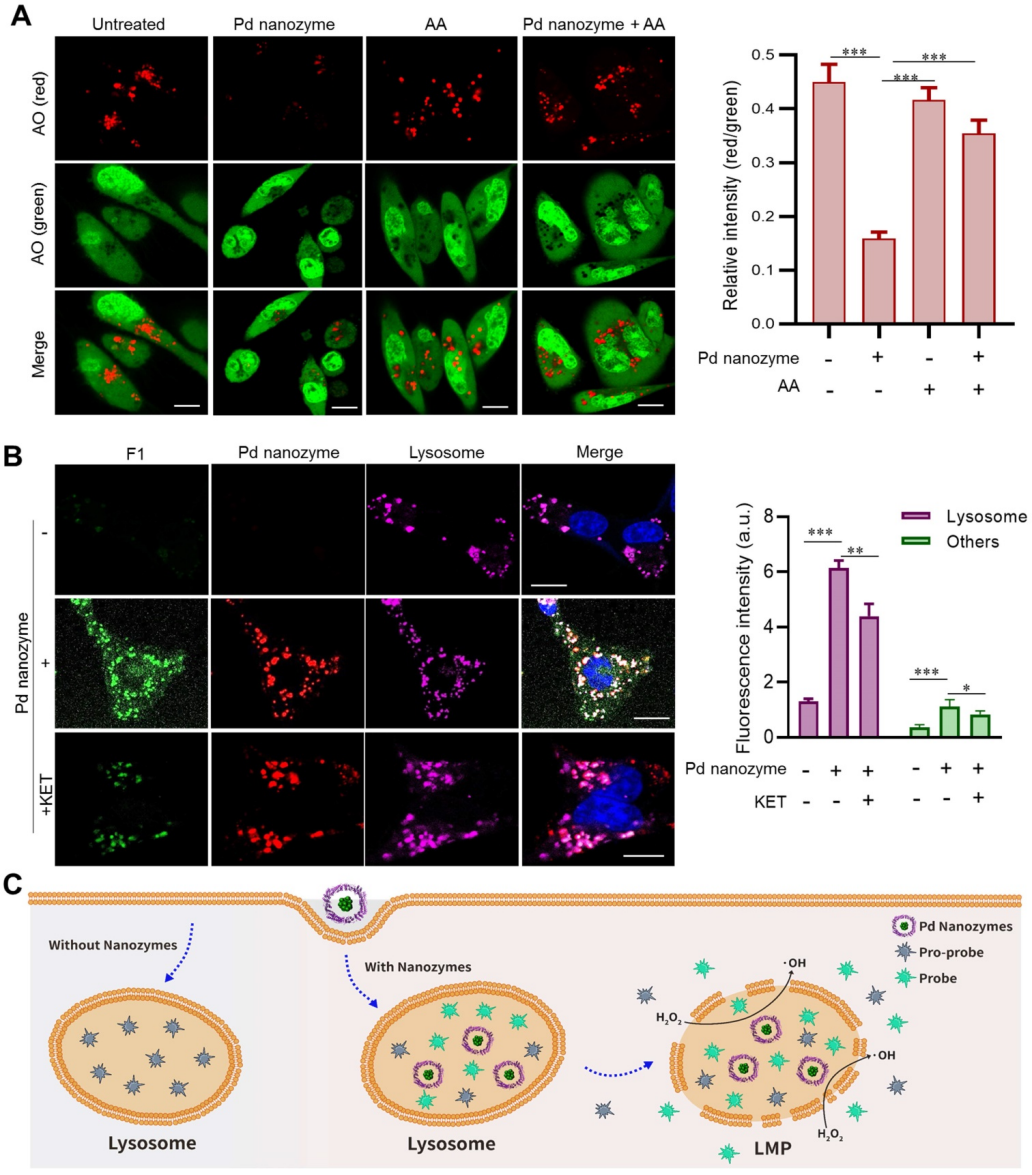
** Pd nanozyme-mediated lysosomal membrane permeability.** (A) Pd nanozymes induced LMP by generation of free radicals in lysosomal microenvironment. Confocal images (left) and quantification analysis (right) of lysosomal membrane leakage in the absence and presence of Pd nanozymes by staining with AO, respectively. Scale bar = 10 μm. ***P < 0.001. (B) Confocal images and quantification analysis of intracellular distribution of activated F1 in the absence and presence of Pd nanozymes. The signal intensity of activated F1 in lysosome and other cellular components were quantified, respectively. *P < 0.05, **P < 0.01, ***P < 0.001. Scale bar = 10 μm. (C) Schematic illustration of the potential underlying mechanism of Pd nanozyme-mediated lysosomal bioorthogonal chemistry. Following active cell uptake, Pd nanozymes were trapped into lysosomes of tumor cells. The P450_BM3_-like activity of Pd nanozymes facilitated the activation of pro-drugs within lysosomes. Furthermore, the generated free radicals by POD-like activty of Pd nanozymes induced lysosomal membrane leakage, resulting in the diffusion of drugs to target effector cell organelles. In contrast, in the absence of Pd nanozymes, the pro-drugs trapped into lysosomes were not activated.

Our previous studies demonstrated FTn protein nanocarriers were capable of active uptake by cancer cells via receptor-mediated endocytosis (i.e. TfR1) 32, 39. By evaluating co-localization of FTn and lysosomes, we first showed that FTn was trafficked into lysosomes (~pH 5.0) after uptake by MDA-MB-231 breast cancer cells, and remained in lysosomes for at least 16 h, as calculated by determining the values of the Pearson's Coefficient (**Figure S6A**). The results revealed that Pd nanozymes persistently remained in lysosomes, an acid microenvironment. Due to Pd nanozymes possessing high POD- and mutant P450_BM_-like activities at pH 5.0 (**Figure 3**), we next sought to explore their synergistic role in lysosomal membrane leakage and *in situ* bioorthogonal catalysis for the release of probes/drugs to cytoplasm. As a classical reaction, POD catalyzes H_2_O_2_ to produce free radicals (i.e. ·OH). By using HPF as a ·OH specific indicator, we also confirmed the ·OH generation ability of Pd nanozymes, as evidenced by the HPF signal increase as the elevation of Pd nanozymes (**Figure S6B**). Previous reports demonstrated that free radicals lead to LMP by inducing lipid damage 40, 41. We thus evaluated whether Pd nanozymes induced lysosomal membrane leakage by staining with AO 42, which produces red fluorescence when accumulated within acidic lysosomes, but dissipates throughout the cytoplasm as green fluorescence in response to LMP. Compared to untreated tumor cells, an obviously reduced signal in lysosomes (red) was observed after treatment with Pd nanozymes. Image-based quantification revealed that the ratio of red to green in untreated- and Pd nanozymes-treated cells were 0.45 and 0.16, respectively (*P* < 0.001) (**Figure 4A**). Furthermore, we studied whether AA, the inhibitor for POD activity, reduced the LMP by simultaneously adding Pd nanozymes and AA into cells. Confocal images and image-based quantification analysis revealed that the LMP induced by Pd nanozymes was significantly suppressed after treatment with AA (*P* < 0.001). These results revealed that Pd nanozymes induced the LMP by producing free radicals within lysosomes.

We then probed *in situ* catalytic activity of Pd nanozymes inside lysosomes. The MDA-MB-231 cells were incubated with or without the nanozyme for 4 h and subsequently washed to remove adsorbed particles before incubation with F1 in fresh medium for a further 4 h. Confocal images showed that there was no obvious signal change in cells absent of Pd nanozymes (**Figure 4B**). In contrast, the cells treated with Pd nanozymes had bright punctate fluorescence, which co-localized with lysosomes. Importantly, we also found that after treatment with Pd nanozymes, the fluorescence signal distributed throughout the whole cell, including cytoplasm and nucleus. Quantification analysis revealed the signal intensity of lysosomes and other cell compartments treated with Pd nanozymes were elevated ~7.7-fold and ~4.1-fold compared to the cells without Pd nanozymes, respectively. To further confirm F1 bond breakage caused by mutant P450_BM_-like activity, the KET inhibitors were added to incubate with the cells following cell uptake of Pd nanozymes. Compared to Pd nanozymes treatment alone, the signal intensity in different organelles (i.e. lysosomes and other cell compartments) was obviously declined after treatment with both Pd nanozymes and KET. Taken together, the lysosomal bioorthogonal chemistry induced by Pd nanozymes could be summarized as follows (**Figure 4C**): following cell uptake, the Pd nanozymes were trafficked into lysosomes; the acidic microenvironment within lysosomes (~pH 5.0) resulted in ·OH production by intrinsic POD-like catalysis of intracellular H_2_O_2_; which in turn induced LMP by destroying lysosomal membrane lipids. In parallel, the mutant P450_BM3_-like activity of Pd nanozymes *in situ* cleaved the propargylic ether bonds of caged small molecules. The activated molecules then penetrated the damaged lysosomal membranes and were subsequently distributed throughout the whole cell by passive diffusion.

### Pd nanozymes-mediated bioorthogonal chemistry

To confirm Pd nanozymes-mediated bioorthogonal chemistry and further expand upon its potential applications, we studied whether the deprotection of propargyl ether by Pd nanozymes was a general strategy for all the corresponding compounds. To do this, additional compounds containing propargyl ether (compounds 1'-4') were synthesized and the structures were confirmed by nuclear magnetic resonance (NMR) spectroscopy (**Figure S7**). After incubation of the synthesized compounds with Pd nanozymes, the products were analyzed by HPLC. The results showed that with the addition of Pd nanozymes, the propargyl ether groups were efficiently cleaved from the compounds, and the conversion efficiency at the same reaction conditions were 88%, 39%, 61% and 76% for compounds 1'-4', respectively (**Figure S8**). These cleavage reactions could possess great therapeutic potential as a general strategy to reduce adverse effects in cancer therapy 43.

**Figure 5:**
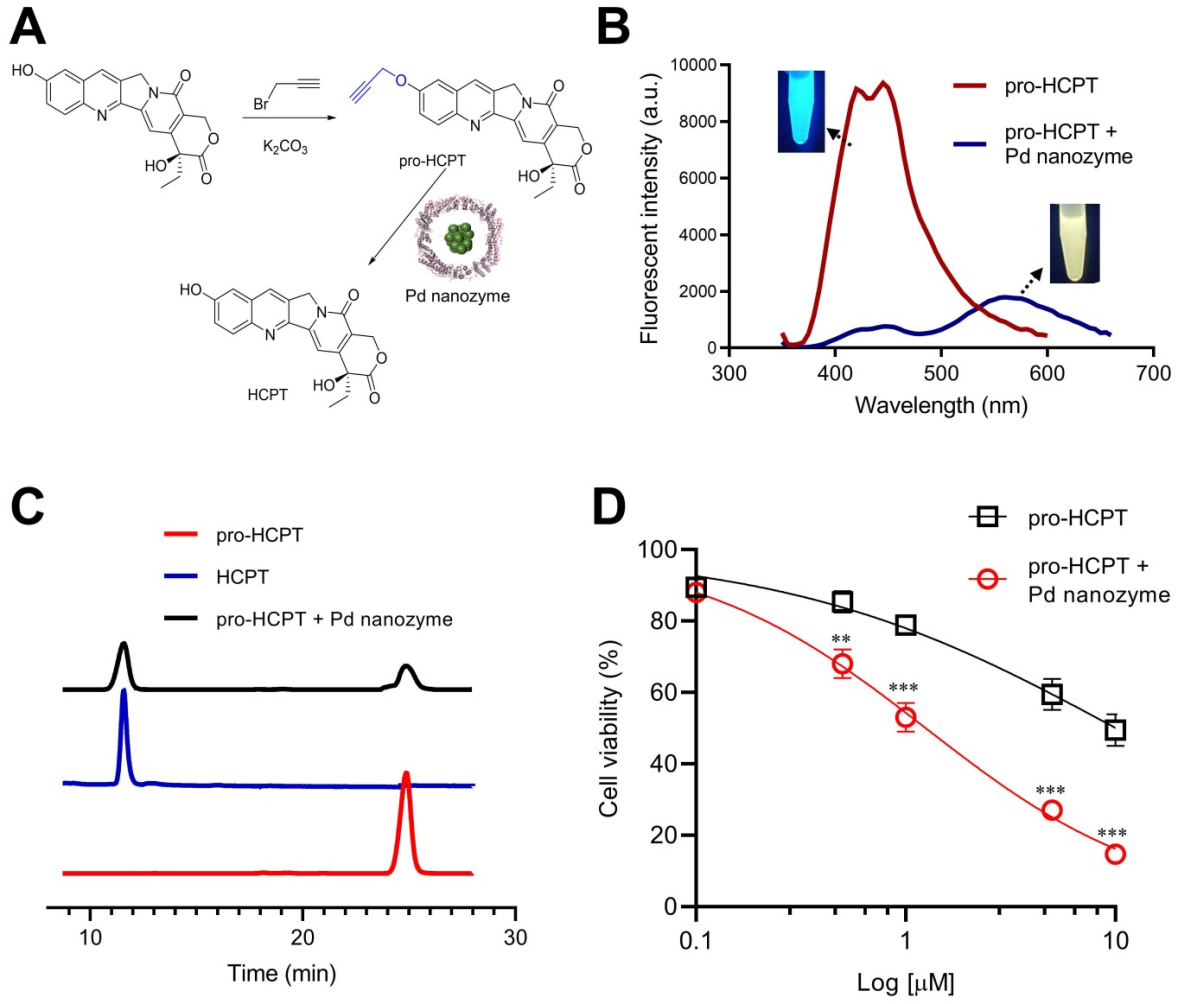
** The deprotection effect of Pd nanozymes on the pro-HCPT.** (A) Schematic illustration of deprotection of the pro-HCPT using Pd nanozymes. (B) The shift of fluorescent emission peak of the pro-HCPT after Pd nanozyme catalysis. (C) HPLC analysis of the cleavage of the propargyl ether from pro-HCPT after incubation with Pd nanozymes. (D) Improved inhibition effect of pro-HCPT on tumor cells in the presence of Pd nanozymes. **P < 0.01, ***P < 0.001.

On the basis that Pd nanozymes could localize in lysosomes after tumor cell uptake and cleave compounds containing propargyl ether *in situ*, we sought to explore whether Pd nanozyme/propargyl ether pairings could be designed for anti-cancer lysosomal pro-drugs. As a model chemotherapeutic drug, hydroxycamptothecin (HCPT) was conjugated with a propargyl ether protecting group (pro-HCPT) by incorporation into the phenolic-hydroxyl groups of the parent drug (**Figure 5A**). The molecular structure and fluorescent peak changes confirmed successful conjugation (**Figure S9**). The deprotection effect of Pd nanozymes on the pro-HCPT was further evaluated. As shown in **Figure 5B**, Pd nanozymes treatment resulted in the shift of fluorescent emission peak of the pro-HCPT from 450 nm to 560 nm, consistent with the spectrum of parent HCPT. Furthermore, with the addition of Pd nanozymes, HPLC analysis confirmed the efficient cleavage of the propargyl ether from pro-HCPT by characterization of the retention time in column (**Figure 5C**). We next tested the ability of Pd nanozymes to activate chemotherapeutic pro-drugs and selectively enhance their potency. The MDA-MB-231 cells were first incubated with Pd nanozymes at a protein concentration of 160 nM (Pd equivalent, 35 μM) for 4 h. After multiple washings, the cells were then incubated with different concentrations of pro-HCPT. As expected, the cells incubated with Pd nanozymes and pro-HCPT showed obviously elevated toxicity compared to that of pro-HCPT only (**Figure 5D**). These results implied the successful intracellular conversion of pro-HCPT into HCPT in the presence of Pd nanozymes. We also confirmed that Pd nanozymes did not substantially affect cell viability including different normal and tumor cell lines, even at higher concentrations and longer incubation time (**Figure S10**). In contrast, Pd^2+^ was able to induce severe cytotoxicity when incubated at lower concentrations (> 13 μM). The results demonstrated that, compared to Pd ions, Pd nanoparticles showed obviously lower cytotoxicity. The possible explaination of Pd^2+^ exhibiting a higher degree of cytotoxicty compared to Pd nanozymes is that Pd^2+^ ions freely diffuse into the cell according to concentration gradients, which allowed Pd^2+^ ions to be distributed throughout the enitiry of the cell. In contrast, Pd nanozymes were mainly localized in lysosomes. Thus, we speculated that lysosomal sequestration reduced nanoparticles availability to other cellular components and decreased the cytotoxicity of nanoparticles.

### Incorporation of nanozymes and pro-drugs into a liposome delivery system

**Figure 6:**
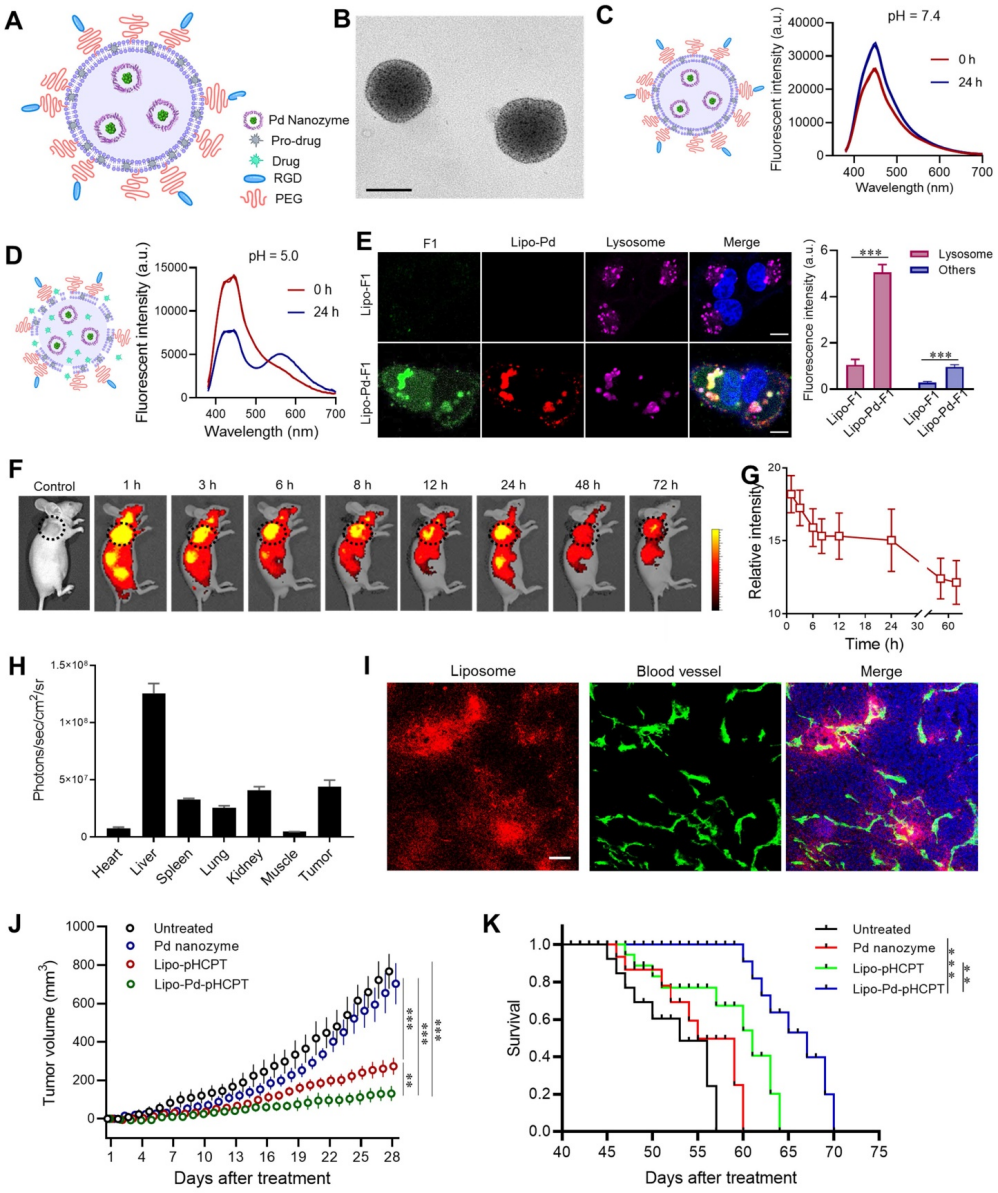
** Liposome-based nanoplatform for anti-cancer therapy.** (A) Design of liposome-based nanoplatform by integration Pd nanozymes and Pro-HCPT. (B) TEM image of Lipo-Pd-pHCPT. Scale bar = 100 nm. Fluorescence properties of Lipo-Pd-pHCPT at (C) pH 7.4 and (D) pH 5.0. (E) Confocal images and quantification analysis of F1 activation in lysosome and other cell components after co-loading with Pd nanozymes into liposome. ***P < 0.001. Scale bar = 10 μm. (F) *In vivo* fluorescent imaging and (G) Image-based quantification analysis of liposome distribution in mice bearing MDA-MB-231 tumor cells (arrow) following i.v. injection over time. (H) *Ex vivo* quantification analysis of biodistribution of liposomes in different organs 72 h after injection. (I) The distribution of liposome-based nanoparticles in tumor tissue after injection. Scale bar = 50 µm. (J) Tumor growth curves of different groups of MDA-MB-231 tumor-bearing mice after various treatments indicated (n = 10 per group). **P < 0.01, ***P < 0.001*.* (K) Kaplan-Meier survival curve of MDA-MB-231 tumor-bearing mice following different treatments. **P < 0.01, ***P < 0.001*.*

The pharmacokinetic difference of Pd nanozymes and pro-drugs limits their *in vivo* targeted drug delivery. Thus, we developed a liposome-based delivery system that combined Pd nanozymes with pro-HCPT (Lipo-Pd-pHCPT). In this “all-in-one” system, the Pd nanozymes were packaged into the inner hydrophilic cavity and the pro-HCPT were integrated into hydrophobic lipid bilayer (**Figure 6A**). The outer surface was decorated with PEG and RGD to provide prolonged *in vivo* blood circulation and active tumor targeting, respectively. TEM images showed that Lipo-Pd-pHCPT has uniform diameters with ~100 nm, and Pd nanozymes (black particles) were embedded into liposome (**Figure 6B**). The loading efficiency of pro-HCPT was calculated as approximately 55% by determining the quantity of pro-HCPT within liposomes. To investigate the stability of Lipo-Pd-pHCPT, we compared the activation of the pro-HCPT at different pH. As shown in **Figure 6C**, the fluorescent emission peak of the Lipo-Pd-pHCPT was not changed when incubated at pH 7.4 buffer for 24 h. However, an obvious peak shift from 450 nm to 560 nm was observed upon lowering the pH to 5.0 (**Figure 6D**), suggesting the activation of pro-HCPT would follow liposome localization within lysosomes. The activation of pro-HCPT at acidic conditions may be due to: (i) the ease of liposomal degradation at lower pH; and (ii) Pd nanozymes generation of free radicals at pH 5.0 improved the lipid permeability of liposomes. To further confirm the activation of pro-drugs in lysosomes, liposomes loaded with both probe F1 and Pd nanozymes (Lipo-Pd-F1) were used as model systems to evaluate the cellular uptake and the cleavage of propargyl ether groups of Lipo-Pd-F1 in tumor cells. Confocal images revealed that liposomes localized within the lysosomes of tumor cells after cellular uptake (**Figure S11**). The fluorescence signal intensity of tumor cells was negligible in the absence of Pd nanozymes, whereas F1 was activated in tumor cells when co-loaded with Pd nanozymes. The fluorescence signal was mainly distributed in lysosomes (**Figure 6E**). Quantification analysis illustrated that the mean fluorescence intensity of lysosomes and other cell components after treatment with Lipo-Pd-F1 were approximately 5.9-fold and 3.4-fold higher than that of Lipo-F1, respectively.

Before evaluation of the therapeutic effects of Lipo-Pd-pHCPT *in vivo*, we tested whether liposomes could target tumor tissues following i.v. administration of Cy5.5-labeled liposomes. *In vivo* fluorescence imaging and quantification analysis demonstrated the efficient accumulation of the particles in tumor areas (arrow) over time, even at 72 h (**Figure 6F and G**). *Ex vivo* fluorescence imaging revealed that the particles mainly distributed in liver and tumors after 72 h post-tail vein injection (**Figure 6H**). The distribution of liposomes in tumors was further studied, and it was revealed that systemically administrated Cy5-labeled liposomes had crossed the endothelium of tumor blood vessels and had penetrated into tumor tissue (**Figure 6I**). We next sought to study whether Pd nanozymes improved *in vivo* therapeutic effects of pro-HCPT by integration into a single delivery system. To do this, the mice bearing MDA-MB-231 tumors were randomly divided into four groups: untreated, Pd nanozymes, Lipo-pHCPT and Lipo-Pd-pHCPT (equivalent Pd nanozymes and pro-HCPT). Subsequently, tumor growth was monitored over time following systemic administration. As shown in **Figure 6J**, compared to untreated group, the Pd nanozymes showed no statistical differences, but Lipo-pHCPT and Lipo-Pd-pHCPT both significantly inhibited tumor growth. Importantly, the mice treated with Lipo-Pd-pHCPT exhibited a signifcant delay in tumor progression compared with mice that were treated with Lipo-pHCPT. Survival was significantly improved in the mice treated with Lipo-Pd-pHCPT, compared to untreated mice. More importantly, the mice treated with Lipo-Pd-pHCPT demonstrated a significant prolonged survival compared with mice that were treated with Lipo-pHCPT **(Figure 6K)**. Furthermore, H&E staining of major organs suggested that there was no apparent accumulation toxicities or damage to the major organs assessed (**Figure S12A**). In addition, there were no significant changes in body weight in all treatment groups compared to the untreated group (**Figure S12B**), suggesting no marked systemic toxicity of the formulations.

## Conclusions

In this study, we described a nanozyme-mediated *in situ* BUR within lysosomes of living cells. This was achieved by taking advantage of the intracellular localization of nanozymes after active cell uptake and their two kinds of enzyme-mimicking activity, including mutant P450_BM3_ and POD. To the best of our knowledge, this is the first demonstration that Pd nanomaterials possess mutant P450_BM3_-like activity. Based on this mutant P450_BM3_-like activity, a Pd nanozyme/protecting group pairing was successfully determined by catalyst screening of various caged fluorophores and rationally designed transition metal-based nanozyme platform. The generated free radicals by POD-like activity of Pd nanozymes induced lysosomal membrane leakage, resulting in the diffusion of cleavaged molecules to target effector cell organelles. Such a multienzyme synergistic approach provides a proof-of-principle study for overcoming lysosomal drug sequestration and improving drug concentrations in their intended targets following pro-drug activation. One limitation of nanozymes is their tendency to simultaneously mimic multiple enzyme-like activities 44. However, this study represents an example of the advantagous application for nanozymes that possess multienzyme activities. Additionally, unlike membrane-impermeable artificial enzymes, the nanocarriers (i.e. FTn and RGD modified liposome) used in this study were capable of actively binding to their corresponding receptors which are expressed in abudance by tumor cells. The active uptake by tumor cells guarantees the designed pro-drugs to be trafficked into the lysosomes of the living tumor cells, and facilitates the distinction between lysosomes of tumor cells and those of normal cells. Such a property endowed that the Pd nanozymes the capcity to remain within lysosomes to provide a persistent catalytic action without causing cytotoxicity or adverse effects *in vivo*. In summary, our strategy represents a promising new direction that utilizes nanotechnology for drug development through* in situ* catalyzing bioorthogonal chemistry within specific subcellular organelles.

## Supplementary Material

Supplementary figures and table.

## Abbreviations

ROS: reactive oxygen species
BUR: bioorthogonal unmasking reactions
POD: peroxidase
HCPT: hydroxycamptothecin
FTn: ferritin nanocages
TEM: transmission electron microscopy
BCA: bicinchoninic acid
EDS: energy dispersive spectroscopy
XPS: X-ray photoelectron spectroscopy
IPTG: isopropyl b-D-1-thiogalactopyranoside
TMB: tetramethylbenzidine
HPF: hydroxyphenyl fluorescein
HPLC: High performance liquid chromatography
DOPC: 1,2-dioleoyl-sn-glycero-3-phosphocholine
DSPE-PEG_2k_: 1,2-distearoyl-sn-glycero-3-phosphoethanolamine-N-[methoxy(polyethylene glycol)-2000
AO: acridine orange
LMP: lysosomal membrane permeability
AA: ascorbic acid
KET: ketoconazole
MTT: methyl thiazolyl tetrazolium
OCT: optimal cutting temperature
H&E: hematoxylin and eosin
SD: standard deviation
NMR: nuclear magnetic resonance
