# Supplementary material for: Bioorthogonal catalytic nanozyme-mediated lysosomal membrane leakage for targeted drug delivery
Source: Theranostics. 2022 Jan 1;12(3):1132–47. doi: 10.7150/thno.66325 (PMC8771541; doi:10.7150/thno.66325)
Supplement: Supplementary file 1 — Supplementary figures and table. [file thnov12p1132s1.pdf]

Supporting Information

Bioorthogonal catalytic nanozyme-mediated lysosomal membrane leakage for targeted drug delivery

Zhiyuan Sun[1,2], Qiqi Liu[2], Xinyue Wang[2], Jin Wu[2,3], Xueyan Hu[2], Miaomiao Liu[1], Xiangyun Zhang[2], Yonghua Wei[2], Zhijun Liu[2], Hongjiang Liu[2], Rui Chen[4], Fei Wang[5], Adam C. Midgley[2], Aitao Li[5], Xiyun Yan[2,6,7], Yanming Wang[1], Jie Zhuang*[3,6], Xinglu Huang*[2,6]*

[1]College of Pharmacy, and State Key Laboratory of Medicinal Chemical Biology, Nankai University, Tianjin 300350, China.
[2]Key Laboratory of Bioactive Materials for the Ministry of Education, College of Life Sciences, Nankai University, Tianjin 300071, China.
[3]School of Medicine, Nankai University, Tianjin 300071, China.
[4]School of Materials Science and Engineering, Nankai University, Tianjin 300350, China.
[5]State Key Laboratory of Biocatalysis and Enzyme Engineering, Hubei Key Laboratory of Industrial Biotechnology, School of Life Sciences, Hubei University, Wuhan 430062, China
[6]Joint Laboratory of Nanozymes, College of Life Sciences, Nankai University, Tianjin 300071, China.
[7]CAS Engineering Laboratory for Nanozymes, Institute of Biophysics, Chinese Academy of Sciences, Beijing 100101, China.

*To whom correspondence may be addressed. Email: wangyanming@nankai.edu.cn, zhuangj@nankai.edu.cn or huangxinglu@nankai.edu.cn

Materials and Methods

General. All solvents were spectroscopic grade and purified by distillation before use. NMR spectra were recorded on a Bruker AVANCE III 400 (400 MHz) spectrometer with deuterium generation reagent and tetramethylsilane as an internal standard.

Synthesis of F1-F9. We synthesized nine compounds and the detailed routes were shown in Supplementary Scheme S1. All compound structures were characterized by NMR and mass spectra. The detailed synthetic procedures were as follows.

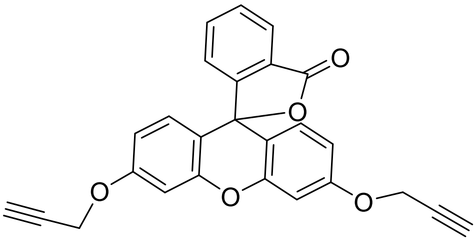

F1

Fluorescein (0.33 g, 1 mmol) and K_2CO_3 (0.27 g, 2 mmol) were dissolved in DMF (5 ml) under stirring. Then propargyl bromide (0.2 ml, 2.5 mmol) were added into the solution. The reaction mixture was reacted at 70 °C for 12 h under nitrogen atmosphere, and then the solvent was removed in vacuum. The crude product was purified by silica gel column chromatography using petroleum ether: ethyl acetate (8:1) as eluent to give the precursor 3',6'-bis(prop-2-yn-1-yloxy)-3H-spiro[isobenzofuran-1,9'-xanthen]-3-one (**F1**) (0.20 g, 49%): ^{1}H NMR (400 MHz, $CDCl_3$) δ (ppm) 2.56-2.57 (m, 2H, ≡CH), 4.72 (d, 4H, J = 4.0 Hz, -CH_2), 6.67-6.73 (m, 4H, phenyl-H), 6.88 (d, 2H, J = 4.0 Hz, phenyl-H), 7.16 (d, 1H, J = 4.0 Hz, phenyl-H), 7.60-7.69 (m, 2H, phenyl-H), 8.02 (d, 1H, J = 4.0 Hz, phenyl-H). MS (ESI):m/z Calcd for $C_{26}H_{16}O_5$ 408.1; found $[M+H]^+$ 409.1.

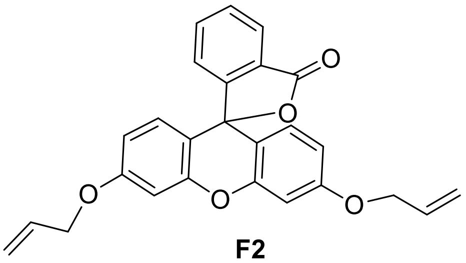

F2

3',6'-bis(allyloxy)-3H-spiro[isobenzofuran-1,9'-xanthen]-3-one (**F2**) was synthesized by similiar methods to **F1**. **F2** (0.28 g, 68%)**:** ^{1}H NMR (400 MHz, $CDCl_3$) δ (ppm) 4.57 (d, 4H, J = 8.0 Hz, =CH_2), 5.32-5.45 (m, 4H, -CH_2), 6.00-6.09 (m, 2H, =CH), 6.61-6.69 (m, 4H, phenyl-H), 6.78 (d, 2H, J = 4.0 Hz, phenyl-H), 7.16 (d, 1H, J = 8.0 Hz, phenyl-H), 7.59-7.68 (m, 2H, phenyl-H), 8.02 (d, 1H, J = 4.0 Hz, phenyl-H). MS (ESI):m/z Calcd for $C_{26}H_{20}O_5$ 412.1; found $[M+H]^+$ 413.1.

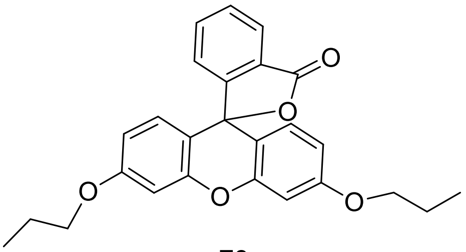

F3

3',6'-dipropoxy-3H-spiro[isobenzofuran-1,9'-xanthen]-3-one (**F3**) was synthesized by similiar methods to **F1**. **F3** (0.29 g, 71 %)**:** ^{1}H NMR (400 MHz, $CDCl_3$) δ (ppm) 1.02-1.06 (m, 6H, -CH_3), 1.79-1.85 (m, 4H, -CH_2), 3.93-3.96 (m, 4H, -CH_2), 6.58-6.61 (m, 2H, phenyl-H), 6.67 (d, 2H, J = 8.0 Hz, phenyl-H), 6.76 (d, 2H, J = 4.0 Hz, phenyl-H), 7.15 (d, 1H, J = 4.0 Hz, phenyl-H), 7.59-7.67 (m, 2H, phenyl-H), 8.02 (d, 1H, J = 4.0 Hz, phenyl-H). MS (ESI):m/z Calcd for $C_{26}H_{24}O_5$ 416.2; found $[M+H]^+$ 417.1.

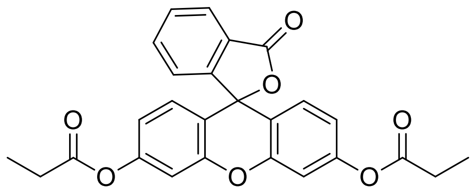

F4

3-oxo-3H-spiro[isobenzofuran-1,9'-xanthene]-3',6'-diyl dipropionate (**F4**) was synthesized by similiar methods to **F1**. **F4** (0.17g, 38%)**:** ^{1}H NMR (400 MHz, $CDCl_3$) δ (ppm) 1.25-1.29 (m, 6H, -CH_3), 2.57-2.63 (m, 4H, -CH_2), 6.79-6.84 (m, 4H, phenyl-H), 7.09 (d, 2H, J = 4.0 Hz, phenyl-H), 7.19 (d, 1H, J = 4.0 Hz, phenyl-H), 7.63-7.69 (m, 2H, phenyl-H), 8.03 (d, 1H, J = 4.0 Hz, phenyl-H). MS (ESI):m/z Calcd for $C_{26}H_{20}O_7$ 444.1; found $[M+H]^+$ 445.1.

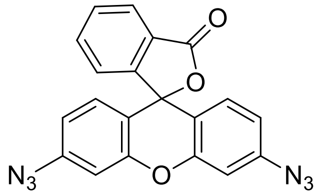

F5

3',6'-diazido-3H-spiro[isobenzofuran-1,9'-xanthen]-3-one (**F5**) was synthesized according to the previous literature [1]. Rhodamine 110 (50 mg, 0.136 mmol) was dissolved in water (4 mL) and HCl (12 N, 1 mL) under nitrogen atmosphere. Solid sodium nitrite (38 mg, 0.551 mmol) was added and the reaction mixture was stirred at room temperature. After 2 h, NaN_3 (53 mg, 0.815 mmol) was added slowly to the above reaction mixture and the reaction was further stirred for 2.5 h. Then, the product was extracted into CH_2Cl_2, washed with brine, and concentrated in vacuo. The crude product was purified by silica gel column chromatography using petroleum ether: ethyl acetate (3:1) as eluent to give **F5** (0.16g, 42%)**.** ^{1}H NMR (400 MHz, $CDCl_3$) δ (ppm) 6.72-6.74 (m, 2H, phenyl-H), 6.78-6.81 (m, 2H, phenyl-H), 6.95 (d, 2H, J = 4.0 Hz, phenyl-H), 7.14 (d, 1H, J = 8.0 Hz, phenyl-H), 7.62-7.71 (m, 2H, phenyl-H), 8.04 (d, 1H, J = 8.0 Hz, phenyl-H). MS (ESI):m/z Calcd for $C_{20}H_{10}N_6O_3$ 382.1; found $[M+H]^+$ 383.1.

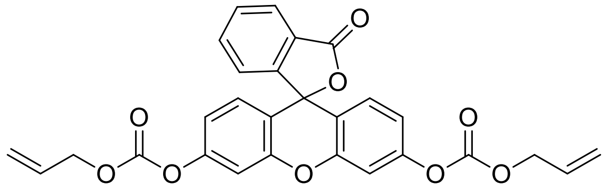

F6

Diallyl (3-oxo-3H-spiro[isobenzofuran-1,9'-xanthene]-3',6'-diyl) dicarbonate (**F6**) was synthesized by similiar methods to **F1**. **F6** (0.15g, 30%)**:** ^{1}H NMR (400 MHz, $CDCl_3$) δ (ppm) 4.75 (d, 4H, J = 4.0 Hz, -CH_2), 5.34-5.46 (m, 4H, =CH_2), 5.95-6.05 (m, 2H, =CH), 6.84 (d, 2H, J = 4.0 Hz, phenyl-H), 6.91-6.93 (m, 2H, phenyl-H), 7.16-7.20 (m, 3H, phenyl-H), 7.64-7.69 (m, 2H, phenyl-H), 8.04 (d, 1H, J = 8.0 Hz, phenyl-H). MS (ESI):m/z Calcd for $C_{28}H_{20}O_9$ 500.1; found $[M+H]^+$ 501.0.

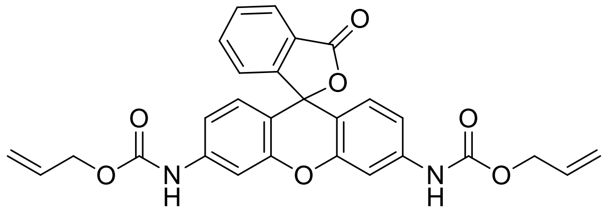

F7

Diallyl (3-oxo-3H-spiro[isobenzofuran-1,9'-xanthene]-3',6'-diyl)dicarbamate (**F7**) (Advanced Drug Delivery Reviews,2017,118,78-93)was synthesized by similiar methods to **F1**. **F7** (0.11g, 20%)**:** ^{1}H NMR (400 MHz, CDCl$_3$) δ (ppm) 4.68 (d, 4H, J = 4.0 Hz, -CH$_2$), 5.27-5.40 (m, 4H, =CH$_2$), 5.93-6.00 (m, 2H, =CH), 6.72 (d, 2H, J = 8.0 Hz, phenyl-H), 6.78 (s, 2H, NH), 6.94-6.97 (m, 2H, phenyl-H), 7.13 (d, 1H, J = 8.0 Hz, phenyl-H), 7.50 (s, 2H, phenyl-H), 7.59-7.68 (m, 2H, phenyl-H), 8.02 (d, 1H, J = 8.0 Hz, phenyl-H). MS (ESI):m/z Calcd for $C_{28}H_{22}N_2O_7$ 498.1; found $[M+H]^+$ 499.1.

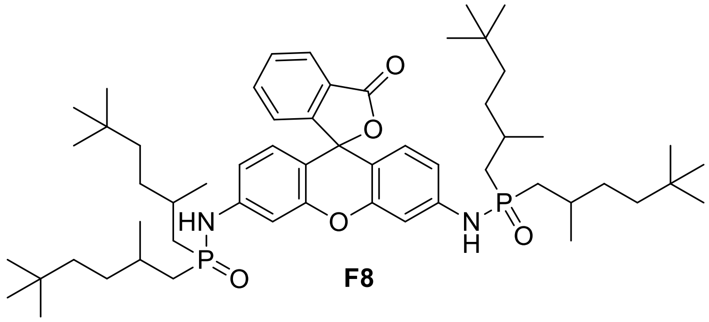

F8

N,N'-(3-oxo-3H-spiro[isobenzofuran-1,9'-xanthene]-3',6'-diyl)bis(P,P-bis(2,5,5-trimethylhexyl)phosphinic amide) (**F8**) was synthesized by similiar methods to **F1**. **F8** (0.12g, 13%)**:** ^{1}H NMR (400 MHz, CDCl$_3$) δ (ppm) 0.88-0.92 (m, 12H, -CH$_3$), 1.12-1.15 (m, 36H, -CH$_3$), 1.22-1.25 (m, 24H, -CH$_2$), 1.28-1.33 (m, 4H, -CH), 6.77 (d, 2H, J = 8.0 Hz, phenyl-H), 7.03-7.06 (m, 2H, -NH), 7.12-7.14 (m, 2H, phenyl-H), 7.34 (d, 2H, J = 4.0 Hz, phenyl-H), 7.52-7.55 (m, 1H, phenyl-H), 7.64-7.68 (m, 2H, phenyl-H), 8.03 (d, 1H, J = 8.0 Hz, phenyl-H). MS (ESI):m/z Calcd for $C_{56}H_{88}N_2O_5P_2$ 930.6; found $[M+H]^+$ 931.1.

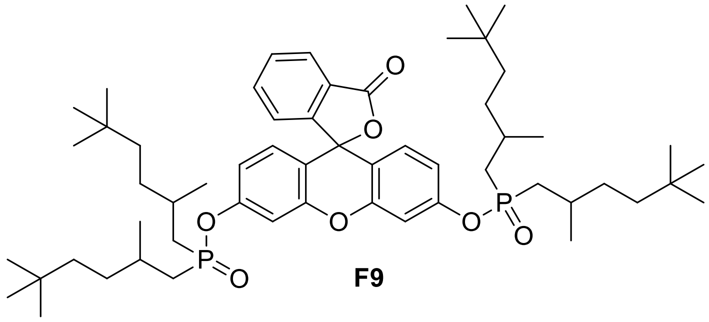

F9

3-oxo-3H-spiro[isobenzofuran-1,9'-xanthene]-3',6'-diyl bis(bis(2,5,5-trimethylhexyl)phosphinate) (**F9**) was synthesized by similiar methods to **F1**. **F9** (0.23g, 25%)**:** ^{1}H NMR (400 MHz, CDCl$_3$) δ (ppm) 0.92 (s, 48H, -CH$_3$), 1.14-1.21 (m, 24H, -CH$_2$), 1.36-1.37 (m, 4H, -CH), 6.77 (d, 2H, J = 8.0 Hz, phenyl-H), 7.03-7.06 (m, 2H, phenyl-H), 7.12-7.14 (m, 1H, phenyl-H), 7.34 (d, 2H, J = 4.0 Hz, phenyl-H), 7.64-7.69 (m, 2H, phenyl-H), 8.04 (d, 1H, J = 8.0 Hz, phenyl-H). MS (ESI):m/z Calcd for $C_{56}H_{86}O_7P_2$ 932.6; found $[M+H]^+$ 933.5.

Synthesis of compound 1'-4'.

Compound **1'-4'** were synthesized by similiar methods to **F1** and according to previous literature [2,3]. The NMR characteration was as follows.

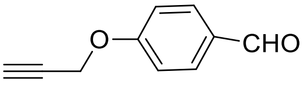

1'

4-(prop-2-yn-1-yloxy)benzaldehyde (1') was synthesized by similiar methods to **F1**. **1'** (0.12g, 75%)**:** ^{1}H NMR (400 MHz, CDCl$_3$) δ (ppm) δ (ppm) 2.56-2.57 (m, 1H, ≡CH), 4.78 (d, 2H, J = 4.0 Hz, -CH$_2$), 7.10 (d, 2H, J = 8.0 Hz, phenyl-H), 7.86 (d, 2H, J = 8.0 Hz, phenyl-H), 9.91 (s, 1H, -CHO).

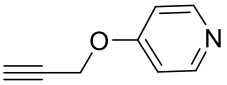

2'

4-(prop-2-yn-1-yloxy)pyridine (2’) was synthesized by similiar methods to **F1**. **2’** (0.09g, 69%)**:** ^{1}H NMR (400 MHz, $CDCl_3$) δ (ppm) δ (ppm) 2.62-2.63 (m, 1H, ≡CH), 4.58 (d, 2H, J = 4.0 Hz, -CH_2), 6.44 (d, 2H, J = 8.0 Hz, pyridyl-H), 7.43 (d, 2H, J = 8.0 Hz, pyridyl-H).

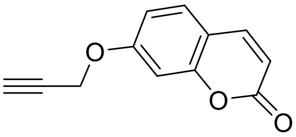

3'

7-(prop-2-yn-1-yloxy)coumarin (3’) was synthesized by similiar methods to **F1**. **3’** (0.14g, 70%)**:** ^{1}H NMR (400 MHz, $CDCl_3$) δ (ppm) δ (ppm) 2.57-2.58 (m, 1H, ≡CH), 4.76 (d, 2H, J = 4.0 Hz, -CH_2), 6.28 (d, 1H, J = 8.0 Hz, coumarin-H), 6.93-6.95 (m, 2H, coumarin-H), 7.40 (d, 1H, J = 8.0 Hz, coumarin-H), 7.64 (d, 1H, J = 8.0 Hz, coumarin-H).

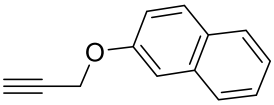

4'

2-(prop-2-yn-1-yloxy)naphthalene (4’) was synthesized by similiar methods to **F1**. **4’** (0.11g, 60%)**:** ^{1}H NMR (400 MHz, $CDCl_3$) δ (ppm) δ (ppm) 2.54-2.55 (m, 1H, ≡CH), 4.81 (d, 2H, J = 4.0 Hz, -CH_2), 7.18-7.21 (m, 1H, naphthyl-H), 7.24-7.26 (m, 1H, naphthyl-H), 7.34-7.38 (m, 1H, naphthyl-H), 7.43-7.47 (m, 1H, naphthyl-H), 7.74-7.77 (m, 3H, naphthyl-H).

Synthesis of pro-HCPT. HCPT (0.36 g, 1 mmol) and K_2CO_3 (0.14 g, 1 mmol) were dissolved in DMF (5 ml) under stirring. Then propargyl bromide (0.12 ml, 1.5 mmol) were added into the solution. The reaction mixture was reacted at room temperature for 12 h under nitrogen atmosphere, and then the solvent was removed in vacuum. The crude product was purified by silica gel column chromatography using petroleum ether: ethyl acetate (3:1) as eluent to give the precursor (4S)-4-ethyl-4-hydroxy-9-(prop-2-yn-1-yloxy)-5,5a-dihydro-1H-pyrano[3',4':6,7]indolizino[1,2-b]quinoline-3,14(4H,12H)-dione.

pro-HCPT (0.10g, 25%)**:** ^{1}H NMR (400 MHz, $CDCl_3$) δ (ppm) 1.02-1.06 (m, 3H, -CH_3), 1.86-1.94 (m, 2H, -CH_2), 2.60 (s, 1H, ≡CH), 3.76 (s, 1H, -OH), 4.86 (d, 2H, J = 4.0 Hz, -CH_2), 5.27-5.32 (s, 2H, -CH_2), 5.29 (d, 1H, J = 20.0 Hz, -CH_2), 5.75 (d, 1H, J = 20.0 Hz, -CH_2), 7.30 (s, 1H, pyridyl-H), 7.50-7.53 (m, 1H, phenyl-H), 7.63 (s, 1H, phenyl-H), 8.15 (d, 1H, J = 8.0 Hz, phenyl-H), 8.29 (s, 1H, pyridyl-H). ^{13}C NMR (400 MHz, $CDCl_3$) δ (ppm) 7.88, 31.65, 50.11, 56.22, 66.41, 97.13, 99.26,107.18, 118.21, 123.66, 129.19, 129.73, 131.45, 145.38, 147.81, 150.23, 151.77, 156.80, 161.78, 174.02. MS (ESI):m/z Calcd for $C_{23}H_{18}N_2O_5$ 402.1216; found $[M+H]^+$ 403.1284.

Reference

1. Sasmal PK. Carregal-Romero S, Han AA, Streu CN, Lin ZJ, Namikawa K, et al. Catalytic azide reduction in biological environments. ChemBioChem. 2012; 13: 1116–20.
2. Alam F, Khan M, Ateeq M. Synthesis of triazole-based nonionic surfactants for nanostructured drug delivery: investigation of their physicochemical and biological aspects. J Surfactants Deterg , 2019; 22(6): 1419-27.
3. Alireza M, Laleh F, Hamid N, Zeinab H, Moghadam FH, Bahar P, et al. Synthesis, docking study, and biological evaluation of novel umbellipherone/hymecromone derivatives as acetylcholinesterase/butyrylcholinesterase inhibitors. Med Chem Res, 2018; 1-7

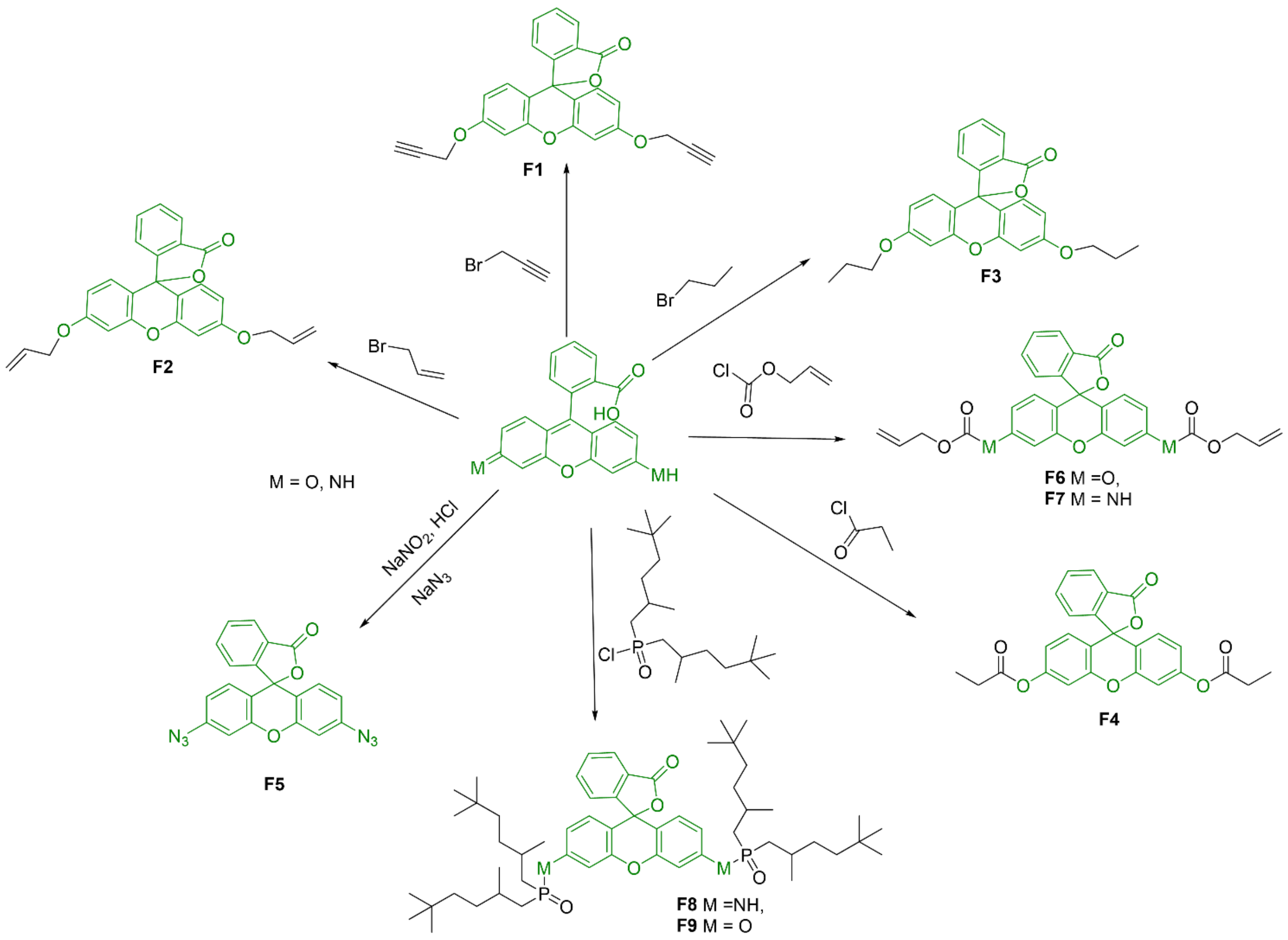

Scheme S1. The detailed routes of compound F1-F9 synthesis.

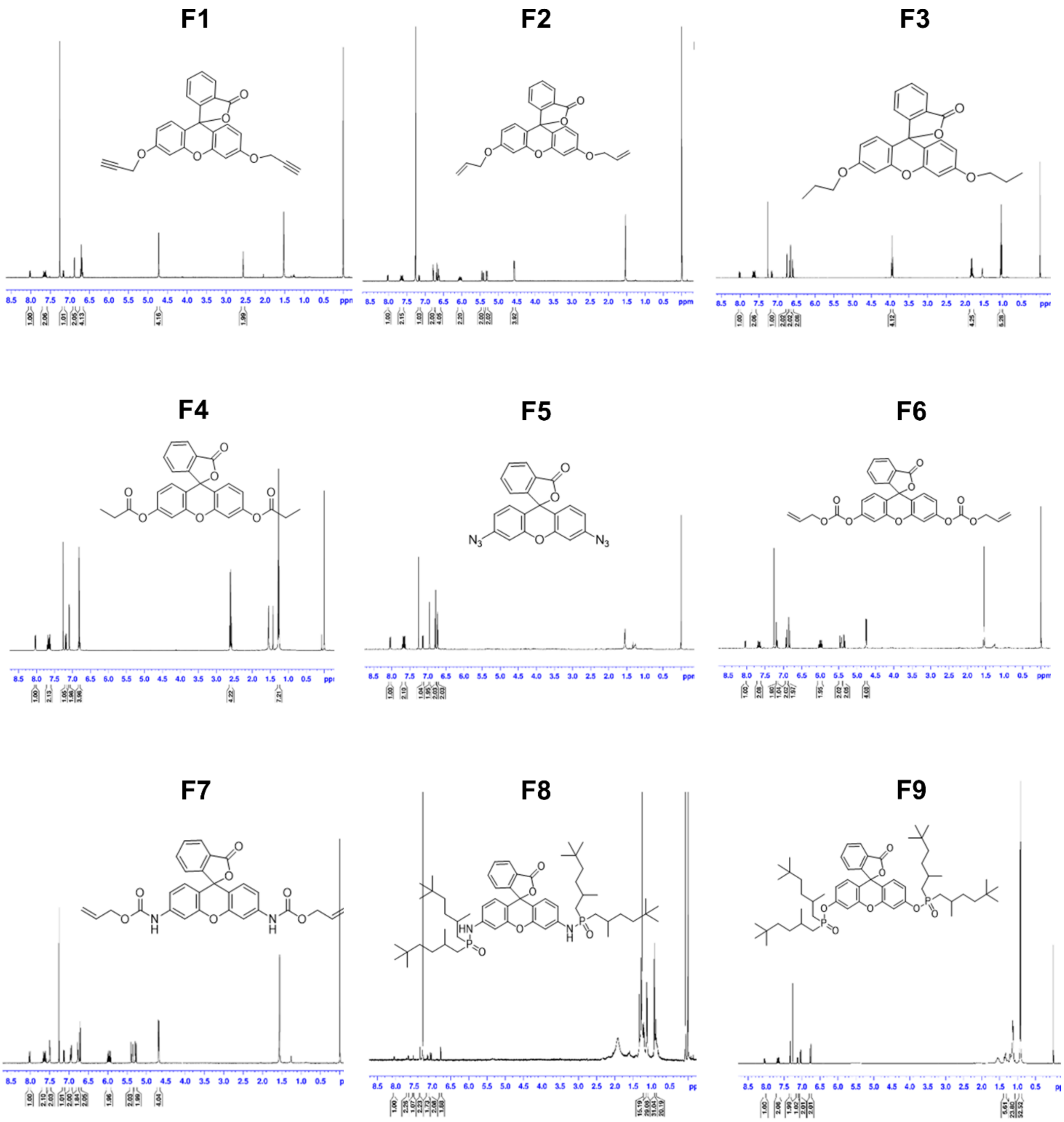

Figure S1. **^{1}H NMR characterization of compound F1-F9.**

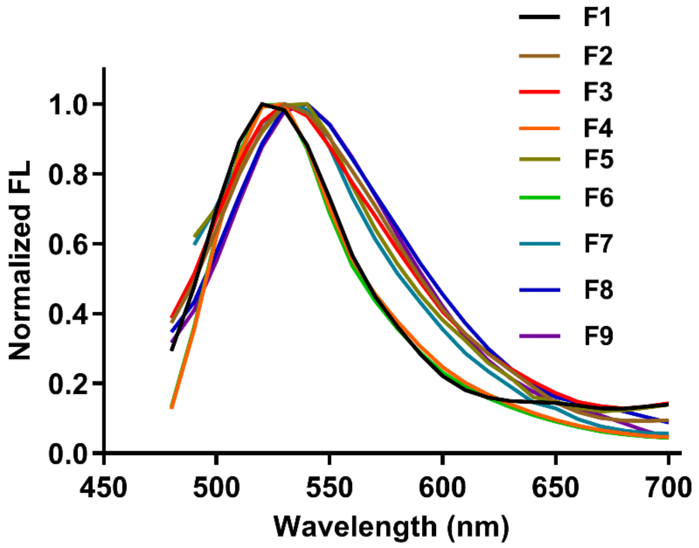

Figure S2. Fluorescence emission spectra of caged fluorophores F1-F9.

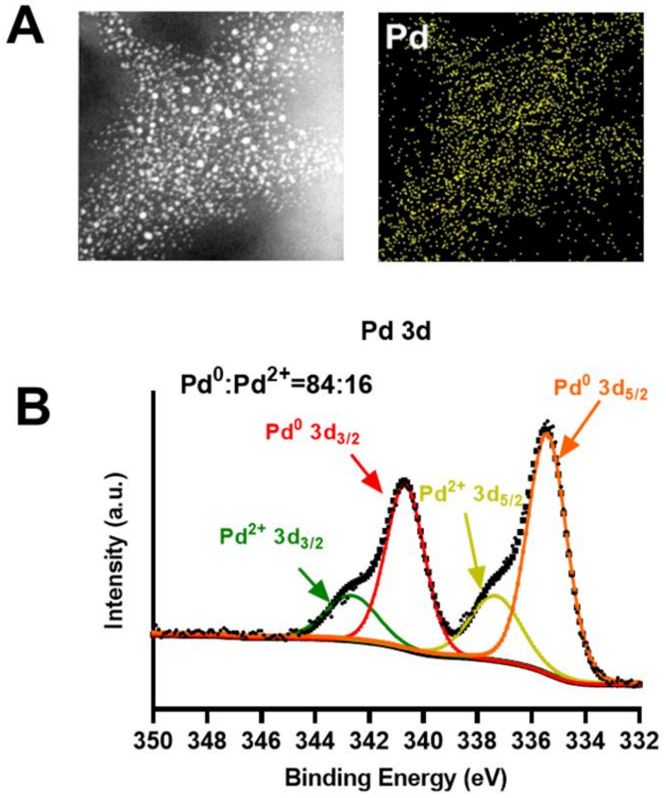

Figure S3. Characterizations of the Pd nanozymes. (A) EDS elemental mapping and (B) XPS spectra of Pd nanozymes.

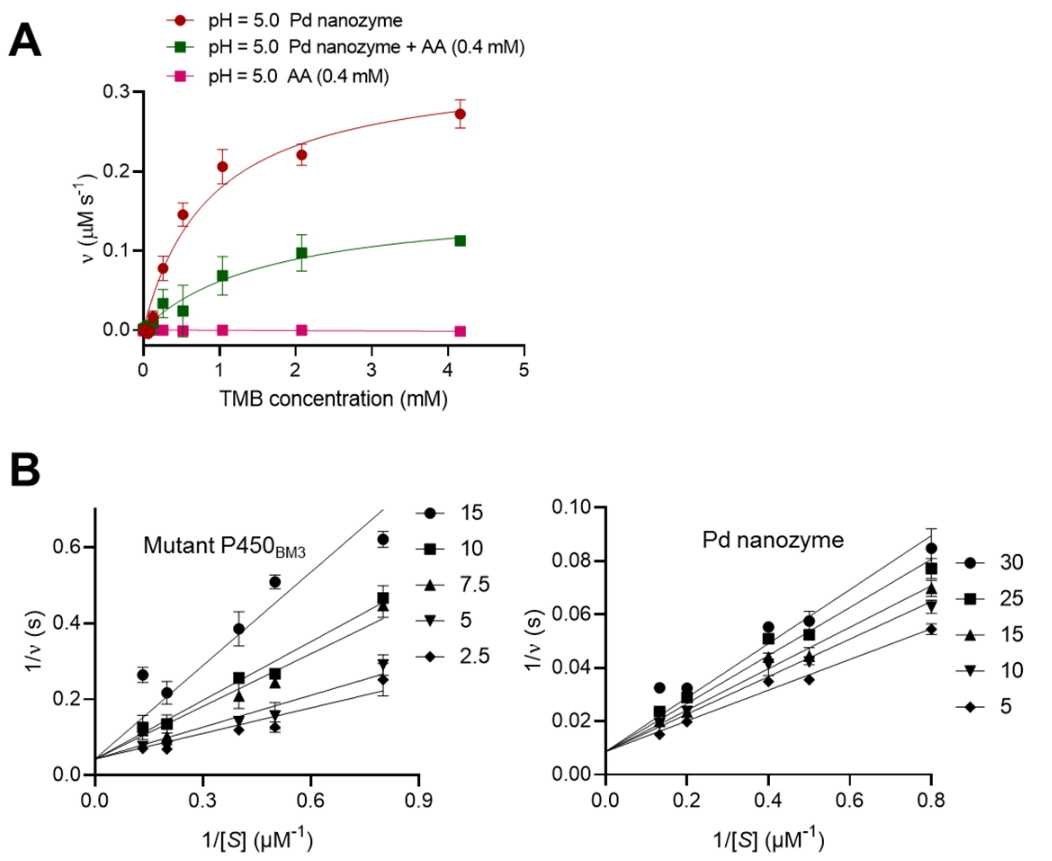

Figure S4. Inhibition of enzymatic activities after treatment with different inhibitors. (A) POD-like activity of Pd nanozymes at pH 5.0 without and with AA, an inhibitor of native POD. (B) The competitive inhibition effect of a $P450_{BM3}$ inhibitor (KET) on Pd nanozymes and mutant $P450_{BM3}$ by determining double reciprocal plots of initial velocity *versus* the substrate (F1) concentration.

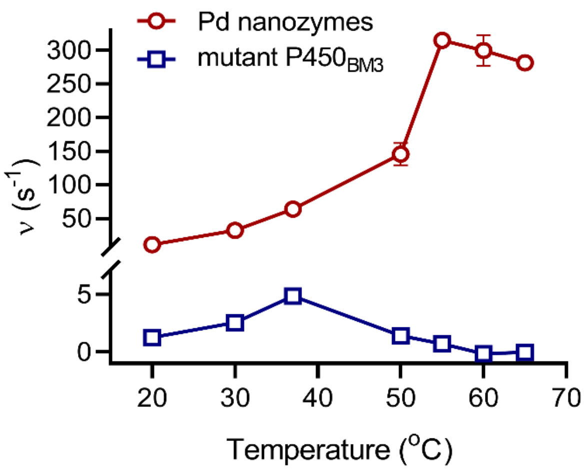

Figure S5. Enzymatic activity comparison of Pd nanozymes and mutant P450$_{BM3}$ towards F1 at different temperature.

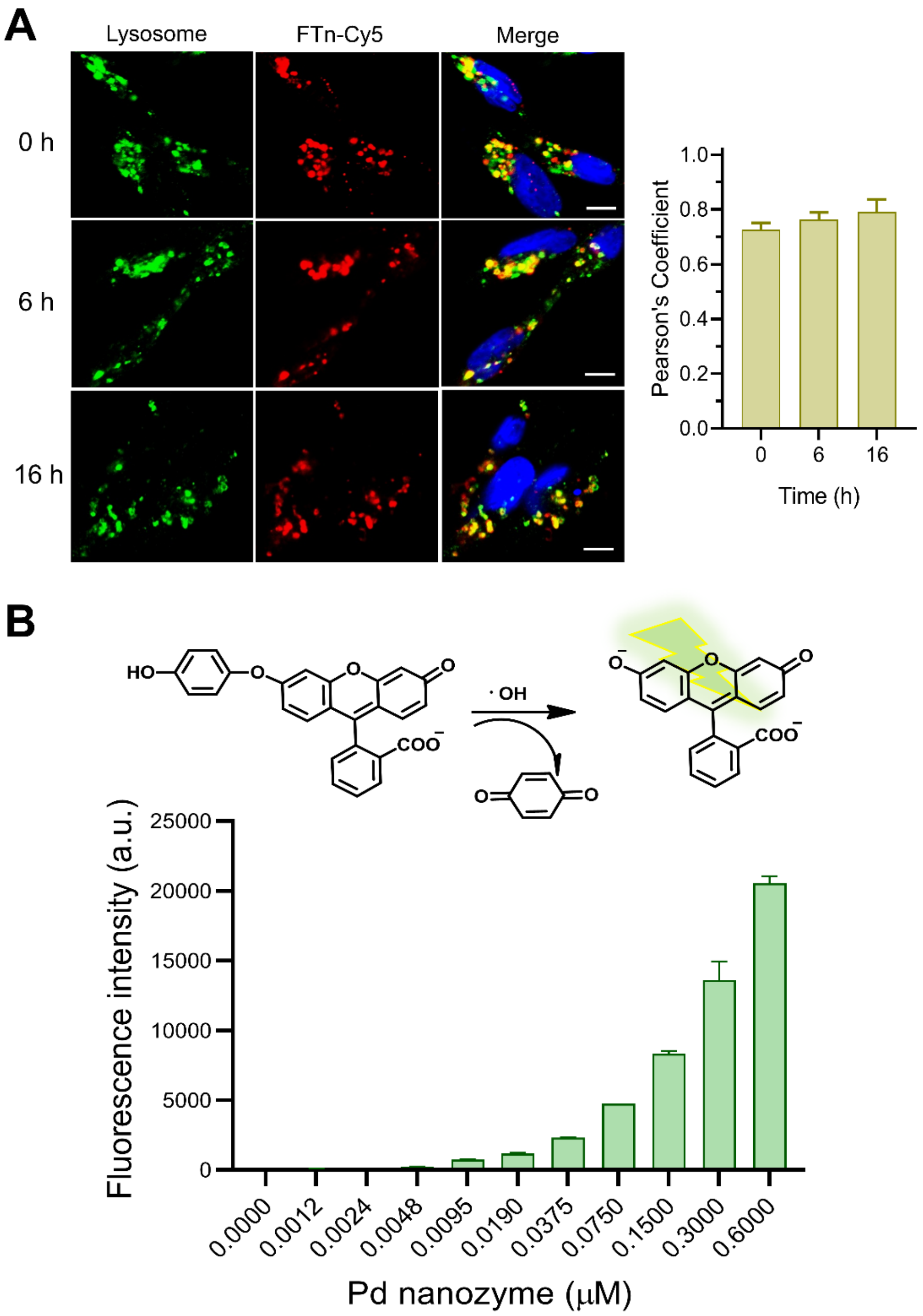

Figure S6. Intracellular distribution of FTn and free radical production of Pd nanozymes. (A) Confocal images (left) and quantification analysis (right) of the colocalization between FTn and lysosome after uptake at different time points. Scale bar = 10 μm. (B) •OH production of different concentration of Pd nanozymes using HPF as an indicator.

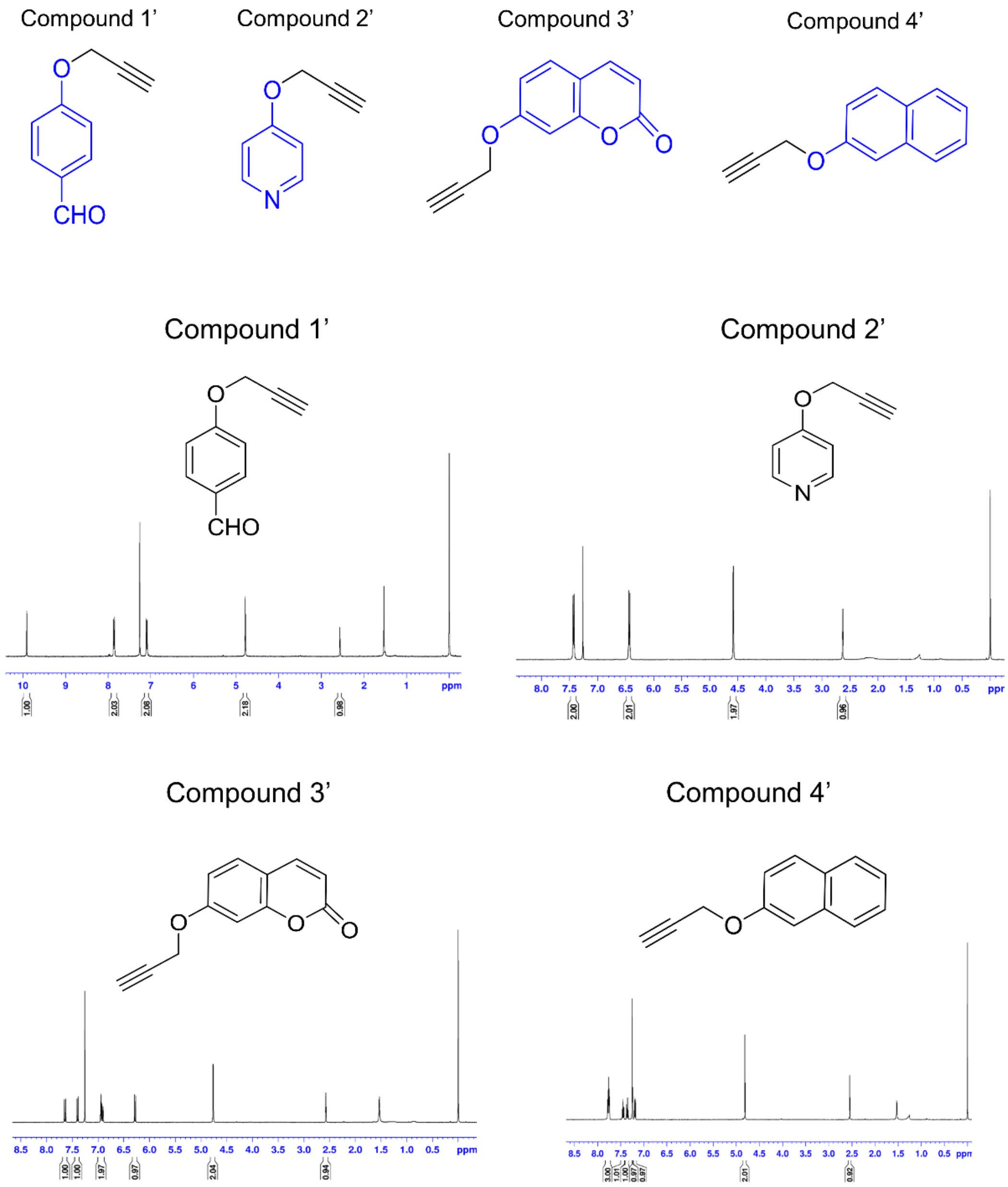

Figure S7. ^{1}H NMR characterization of compound 1'-4'.

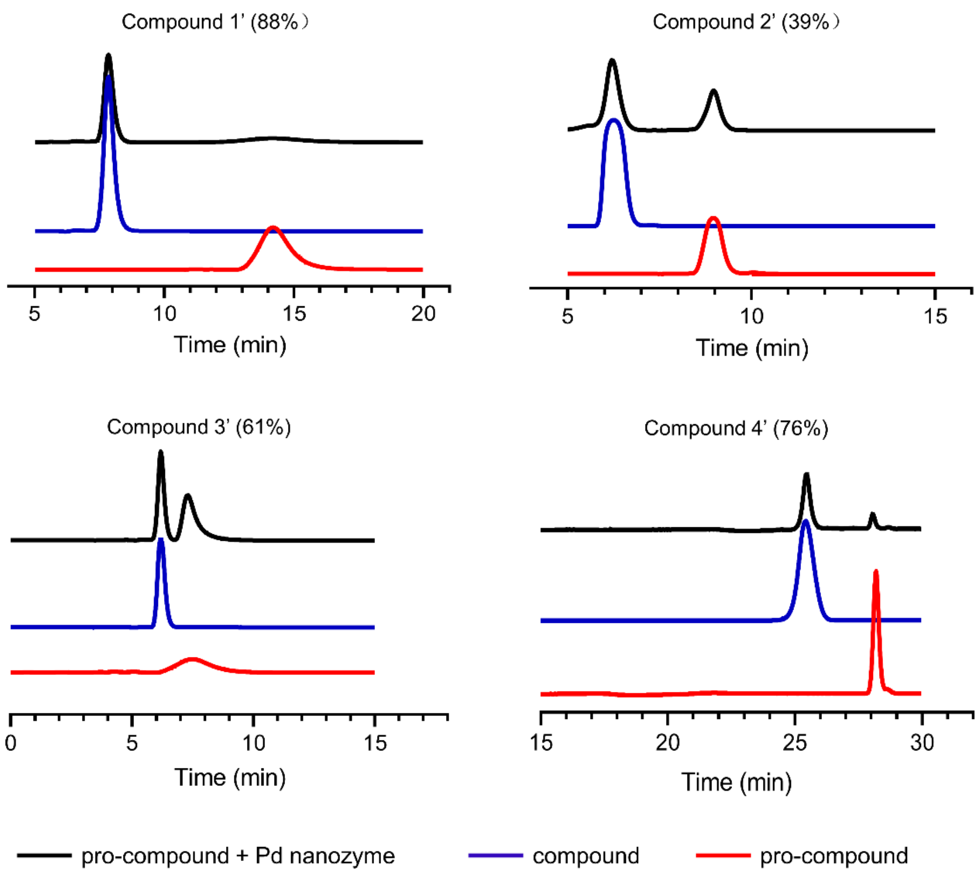

Figure S8. HPLC characterization of compound 1'-4' deprotecting by Pd nanozymes. The cleavage efficiency of the propargyl ether were 88%, 39%, 61% and 76% for compound 1'-4', respectively.

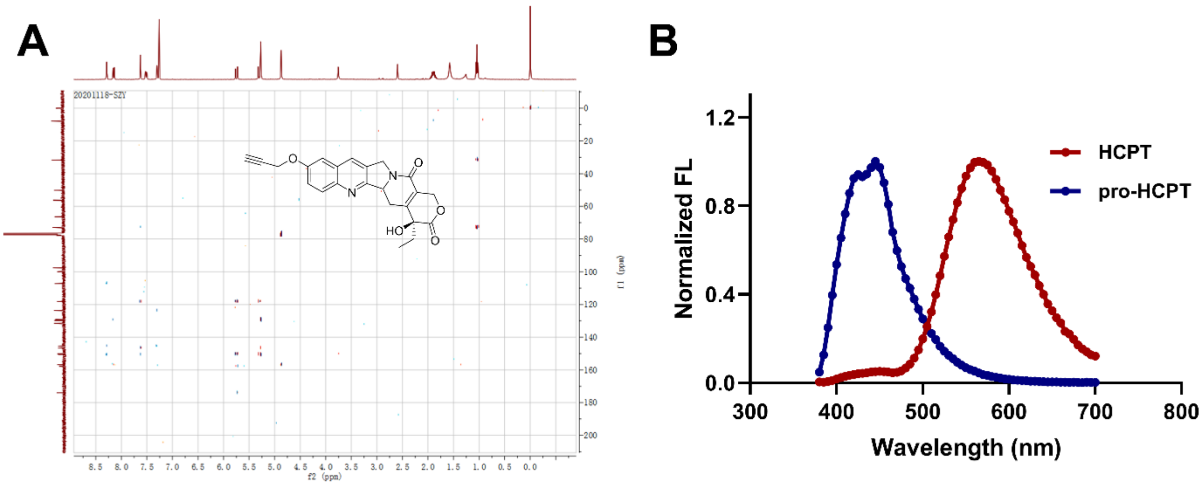

Figure S9. **^{1}H NMR characterization (A) and fluorescence property (B) of pro-HCPT.**

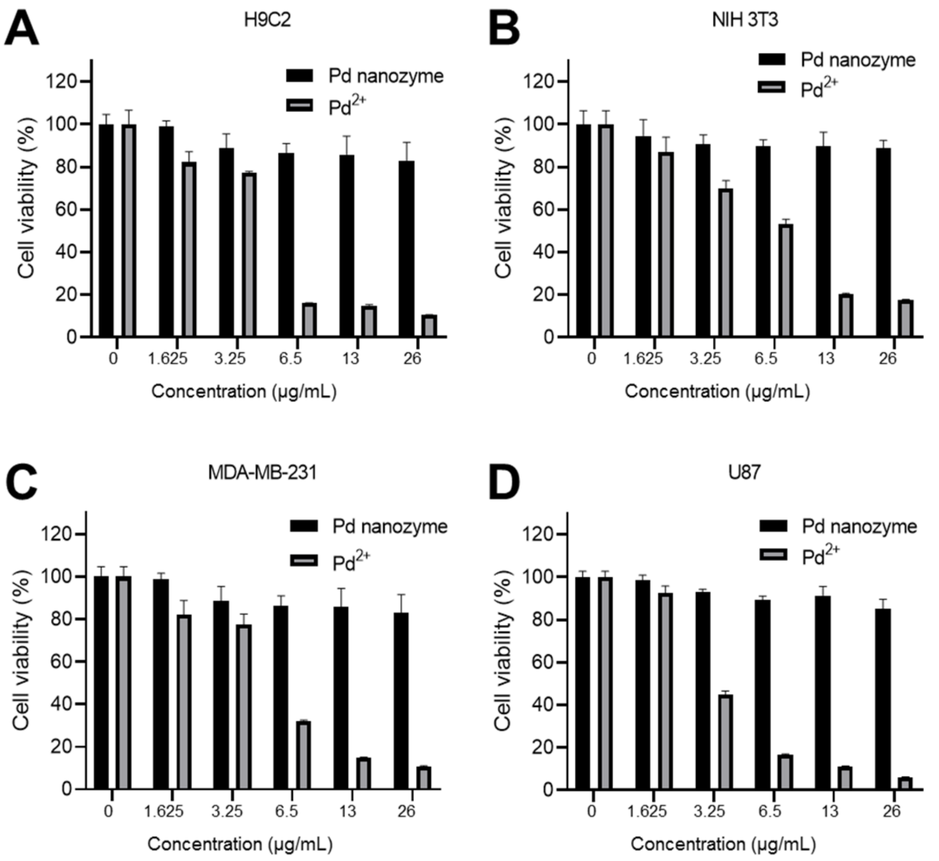

Figure S10. The cytotoxicity of Pd nanozymes and $PdCl_2$ with same concentrations of Pd element on cell viability. Pd elements involved in Pd nanozymes were characterized by ICP-MS. For Pd nanozymes, 1 mg/mL FTn concentration was equivalent with 0.212 mg/mL Pd elements. H9C2, cardiac myoblast (A); NIH 3T3, embryonic fibroblast cells (B); MDA-MB-231, breast cancer cells (C) and U87, glioblastoma cells (D).

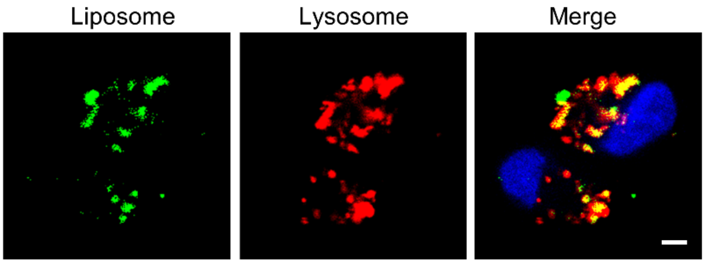

Figure S11. Confocal images of colocalization of liposome and lysosome. Scale bar = 10 μm.

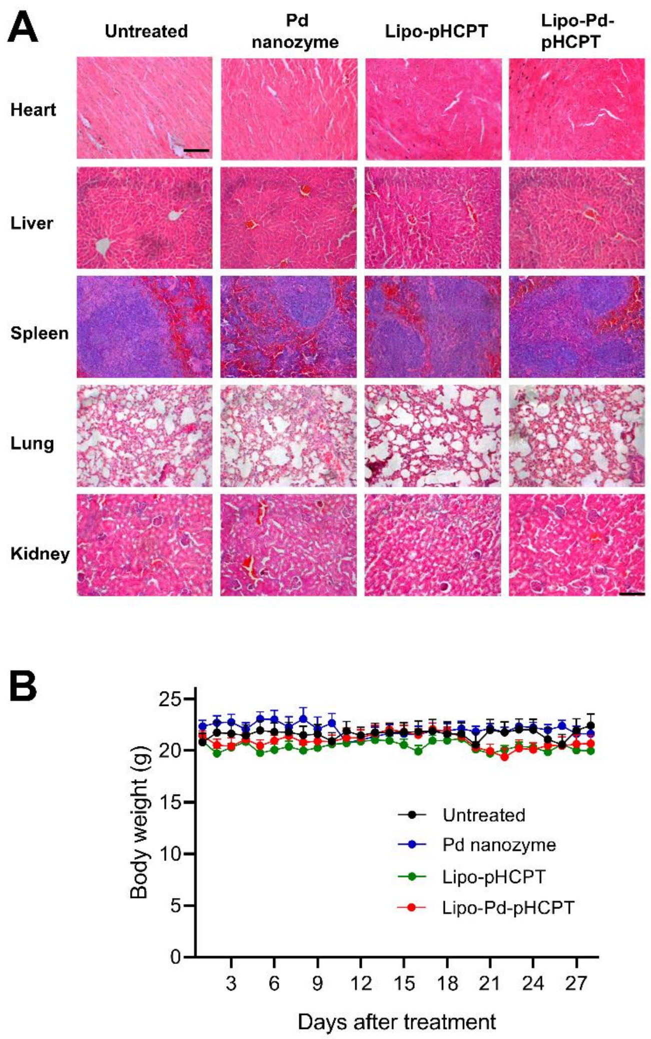

Figure S12. *In vivo* toxicity analysis of mice with different treatments. (A) *In vivo* hematological analysis of systemic toxicities in different organs isolated from the mice after indicated treatments. (B) The effect of different treatments on mice body weight. Scale bar = 100 μm.

Table S1

	$[E]$ (μM)	V_{max} (s^{-1})	K_m (μM)	k_{cat} ($\mu M^{-1}\ s^{-1}$)	k_{cat} / K_m
Pd nanozyme	0.15	126.6	4.049	844	208
Mutant $P450_{BM3}$	0.3	4.546	2.971	15.15	5

K_m: Michaelis constant, the substrate concentration at which the initial rate is half of the maximum velocity.
k_{cat}: turnover number, the catalytic constant for the conversion of substrate to product.
